# SignifiKANTE: efficient *P*-value computation for gene regulatory networks

**DOI:** 10.1093/bioinformatics/btag531

**Published:** 2026-07-22

**Authors:** Fabian Woller, Paul Martini, Souptik Sen, David B Blumenthal, Anne Hartebrodt

**Affiliations:** Biomedical Network Science Lab, Friedrich-Alexander-Universität Erlangen-Nürnberg, Erlangen, Germany; Institute for Biomedicine, Eurac Research, Bolzano, Italy; Biomedical Network Science Lab, Friedrich-Alexander-Universität Erlangen-Nürnberg, Erlangen, Germany; Biomedical Network Science Lab, Friedrich-Alexander-Universität Erlangen-Nürnberg, Erlangen, Germany; Peter L. Reichertz Institut für Medizinische Informatik, Medizinische Hochschule Hannover, Hannover, Germany; Biomedical Network Science Lab, Friedrich-Alexander-Universität Erlangen-Nürnberg, Erlangen, Germany; Biomedical Network Science Lab, Friedrich-Alexander-Universität Erlangen-Nürnberg, Erlangen, Germany

## Abstract

**Motivation:**

Gene regulatory networks (GRNs) are graph-based representations of regulatory relationships between transcription factors and target genes. Various tools exist to infer GRNs from gene expression data, but since GRN inference itself is already computationally intensive, statistical significance estimates are often omitted. While naïve permutation-based empirical *P*-value computation methods are relatively straightforward to implement, they are prohibitively expensive when applied to popular regression-based GRN inference methods and realistically sized datasets.

**Results:**

To address this bottleneck, we developed SignifiKANTE, a tool to efficiently quantify edge significance in GRNs obtained via any regression-based GRN inference method. SignifiKANTE is based on the key insight that the background count distributions of groups of target genes may be highly similar, even if their expression vectors show distinct behavior. Relying on this insight, SignifiKANTE uses gene clustering based on the 1-Wasserstein distance to create a small, constant number of background distributions which enables the simultaneous computation of approximate permutation-based *P*-values for multiple target genes. This reduces the runtime by orders of magnitudes (for some datasets, from several weeks to few hours), without compromising faithfulness of the obtained *P*-values.

**Availability and implementation:**

SignifiKANTE’s Python source code is available at https://github.com/bionetslab/SignifiKANTE, a packaged version at https://pypi.org/project/signifikante, and scripts to reproduce the results at https://github.com/bionetslab/SignifiKANTE_Results.

## 1 Introduction

Gene regulatory networks (GRNs) describe how transcription factors (TFs) (Lambert *et al.* 2018) interact with target genes to control gene expression and enable the complex biological processes that support life. These networks provide key insights into physiological processes, like cell differentiation, disease progression, and response to external stimuli. Since experimentally determining regulatory links between TFs and their targets is expensive and such links can differ substantially between different conditions, tissues, and cell types, GRNs are often reconstructed from a high-throughput bulk or single-cell gene expression dataset $X\in R^{n\times m}$ using computational methods (n denotes the number of samples or cells in the dataset, m denotes the number of genes). Various *in silico* GRN inference methods exist (Pratapa *et al.* 2020). Typically, a TF-target gene edge is inferred between two genes if they are statistically associated across the samples (or cells in the case of single-cell data) in the dataset X. An edge-wise score indicating regulation strength is typically provided as well.

Here, we focus on one of the most common classes of GRN inference methods, which uses linear or non-linear regression to model the task of inferring a GRN from the gene expression data   X (Huynh-Thu *et al.* 2010, Haury *et al.* 2012, Aibar *et al.* 2017). These methods work as follows: Given a set of TFs T⊆{1,…,m} as candidate regulators, a regression model


(1) $$y_{j}∼f_{j}(X_{•,T\setminus \{j\}}),$$

that predicts the expression profile $y_{j}=X_{•,j}$ from the expression profiles of all TFs (except for j itself if j is a TF) is fit for each gene j∈{1,…,m}. Then, feature attribution methods are used that quantify the contributions of the TFs t∈T in the models $f_{j}$. The obtained feature attribution scores are treated as weights w(t,j) of a potential regulatory link from the TF t to the target gene j, which are then aggregated across all targets j∈{1,…,m}. Finally, a GRN containing the links with the largest weights is returned. To make the weights w(t,j) comparable across different target genes, the columns of X are typically normalized to unit variance before fitting the models $f_{j}$ (e.g. using *z*-score normalization) (Huynh-Thu *et al.* 2010). Popular regression-based methods for GRN construction include GENIE3 (Huynh-Thu *et al.* 2010) and GRNBoost2 (Moerman *et al.* 2019). GENIE3 uses random forest or extra tree regressors, which are replaced by gradient boosting with early stopping in GRNBoost2 to improve computational efficiency. Although regression-based methods such as GENIE3 and GRNBoost2 have performed very well in independent benchmarks (Pratapa *et al.* 2020), they share the limitation that they rely on *ad hoc* cutoffs for the weights or the number of regulatory links to decide which edges (t,j) to include in the aggregated GRN. As a consequence, GRNs inferred by regression-based methods tend to include many false-positive edges and have been shown to often lack robustness to random bias (Ketteler and Blumenthal 2023).

To address these issues and reduce the number of spurious interactions in inferred GRNs, statistical approaches to assess the significance of the edge weights w(t,j) are required. For GENIE3, such an approach has been developed as part of the DIANE dashboard for GRN inference and analysis (Cassan *et al.* 2021). In DIANE, empirical *P*-values are computed for weights w(t,j) through repeated shuffling of the target gene expression vectors , followed by random forest fitting with the permuted target vectors ${\tilde{y}}_{j}$. This approach generalizes to all regression-based GRN inference methods (see Algorithm A.1 in Appendix A, available as supplementary data at *Bioinformatics* online) but suffers from severe scalability issues. It requires fitting *k* regression models, where k is the number of permutations determining the *P*-value resolution (typically k≥1000). This is infeasible for GRNs inferred from realistically sized gene expression data with m∈[15 000,20 000]. In DIANE, this problem is mitigated through two aggressive filtering steps prior to *P*-value computation: (i) restricting GRN inference to differentially expressed genes and (ii) applying a hard threshold on the weights w(t,j) to a achieve a user-specified (small) edge density. While these two filters make permutation-based *P*-value computation computationally feasible, they both involve additional hyperparameters without providing a clear rationale or data-driven strategy for their selection.

Further statistical approaches for validating edges in regression-based GRNs have been proposed by Morgan *et al.* (2019) and Wang *et al.* (2024). Morgan *et al*.’s strategy consists of repeated bootstrapping-based dataset resampling to improve stability and reduce false positives across a variety of regression-based GRN inference algorithms, including GENIE3 (Huynh-Thu *et al.* 2010), LASSO (Tjärnberg *et al.* 2015, Schmitt *et al.* 2023), and CLR (Faith *et al.* 2007). Wang *et al.* developed an approach using perturbation-induced data coupled with bootstrapping and consensus-based link inclusion criteria to control the false discovery rate (FDR) in GRNs. However, both approaches have an even higher computational cost than the method implemented in DIANE and have been tested on data containing up to only 45 (Morgan *et al.* 2019) and 102 (Wang *et al.* 2024) genes, respectively.

Beyond approaches that can act on top of any regression-based GRN inference method, Yeh *et al.* (2009) suggested a method that infers GRNs from microarray data through pairwise independence testing followed by conditional independence testing and thus natively provides edge-level *P*-values. The same holds for the mutual information-based methods ARACNE (Margolin *et al.* 2006) and ARACNe-AP (Lachmann *et al.* 2016), which yield edge-level empirical *P*-values by generating one joint background distribution for all TF-target gene pairs. Another related approach is the scNAE framework proposed by Wang and Liu (2024), which uses ordinary differential equations to compute a network-level *P*-value that quantifies overall consistency between an input GRN and a given single-cell RNA-sequencing (scRNA-seq) dataset. However, all of these approaches are tied to specific GRN inference methods and do not generalize to popular regression-based methods such as GENIE3 and GRNBoost2.

With SignifiKANTE (from “signifikante Kante,” German for “significant edge”), we present an algorithm for permutation-based assessment of edge significance in GRNs inferred via any regression-based GRN inference method that overcomes the runtime limitations of existing general-purpose approaches (Morgan *et al.* 2019, Cassan *et al.* 2021, Wang *et al.* 2024) and scales to real-world gene expression data. SignifiKANTE makes critical use of the following key observation: In general, GRN inference methods pick up on statistical associations between two genes across the samples in the dataset. However, in the shuffled data, these statistical associations are removed. As a consequence, two genes with the same count distribution have identical null distributions, even if they are not statistically associated in the original expression data. Exploiting this observation, SignifiKANTE uses the following strategy to efficiently compute *P*-values for all edges in the originally inferred GRN: Instead of fitting one regression model for each of the m target genes for all k permutations, we first compute pairwise 1-Wasserstein distances between the column-normalized count distributions of all genes and use hierarchical clustering to create a constant number ℓ of null distribution prototypes. Based on these clusters, we create a proxy dataset containing representative genes for each cluster. This reduced dataset is then used to compute the null distribution by applying the user’s regression-based GRN inference method of choice. Since ℓ≪m, this substantially speeds-up the computation of *P*-values for all edges in a given GRN. We show that increasing ℓ offers diminishing returns beyond ℓ=10, such that the choice of hyperparameter can be omitted in practice. SignifiKANTE is method-agnostic and compatible with every regression-based GRN inference approach.

To test SignifiKANTE’s efficiency and approximation quality, we compared against exact permutation-based *P*-values as implemented in DIANE, using 30 tissue gene expression datasets from GTEx (GTEx Consortium 2013) and three scRNA-seq datasets from human peripheral blood mononuclear cell (PBMC) samples (De Simone *et al.* 2025). Moreover, we compared GRNs with edges filtered based on SignifiKANTE’s *P*-values to vanilla GRNBoost2, relying on simulated data and comparisons of GRNs inferred from GTEx datasets to known TF-target gene links from CollecTRI (Müller-Dott *et al.* 2023), as well as their contextualization in the light of the BRENDA tissue ontology (BTO) (Gremse *et al.* 2011). SignifiKANTE is implemented in Python as an add-on for the widely used GRN inference package Arboreto (Moerman *et al.* 2019), with out-of-the-box support for GENIE3, GRNBoost2, as well as LASSO and ridge regression-based GRN inference as implemented in GReNaDIne (Schmitt *et al.* 2023). For users interested in using SignifiKANTE with other regression-based GRN inference methods, we provide a tutorial on how to easily integrate additional methods.

## 2 Results

### 2.1 Overview of SignifiKANTE

SignifiKANTE is based on the following key insight: If there are two target genes j and $j^{\prime }$ whose columns $X_{•,j}={\left(x_{i,j}\right)}_{i=1}^{n}$ and $X_{•,j^{\prime }}={\left(x_{i,j^{\prime }}\right)}_{i=1}^{n}$ in the gene expression matrix X are identical up to sample permutation, then one of them is redundant under column-wise shuffling. When computing background GRN edge weights via sample shuffling to obtain DIANE-like empirical *P*-values, we can remove $j^{\prime }$ from the set of targets and use j*’*s in-edges as proxies for those of $j^{\prime }$. We thus call gene expression vectors $X_{•,j}$ and $X_{•,j^{\prime }}$  *background-equivalent* if $\mathrm{sorted}(X_{•,j})=\mathrm{sorted}(X_{•,j^{\prime }})$, where sorted(·) denotes the sorting operation in non-decreasing order. Observation 1 characterizes background-equivalent gene expression vectors in terms of the 1-Wasserstein distance.

Observation 1. *Two gene expression vectors* $X_{•,j}$  *and* $X_{•,j^{\prime }}$  *are background-equivalent if and only if* $W_{1}(X_{•,j},X_{•,j^{\prime }})=0$*, where* $W_{1}(\cdot ,\cdot )$  *is the 1-Wasserstein or earth mover’s distance.*

**Proof.** Since $X_{•,j}$ and $X_{•,j^{\prime }}$ have the same length n and contain 1D data, it holds that

(2) $$W_{1}(X_{•,j},X_{•,j^{\prime }})=\frac{1}{n}\sum_{i=1}^{n}|x_{(i),j}-x_{(i),j^{\prime }}|,$$

where the subscript (i) denotes the $i^{\text{th}}$ order statistic. Since all summands in the right-hand side of Equation (2) are non-negative, $W_{1}(X_{•,j},X_{•,j^{\prime }})=0$ holds if and only if $|x_{(i),j}-x_{(i),j^{\prime }}|=0$ for all i=1,…,n. By definition of the order statistics, this is the case if and only if $\mathrm{sorted}(X_{•,j})=\mathrm{sorted}(X_{•,j^{\prime }})$. ▪

**Figure 1:**
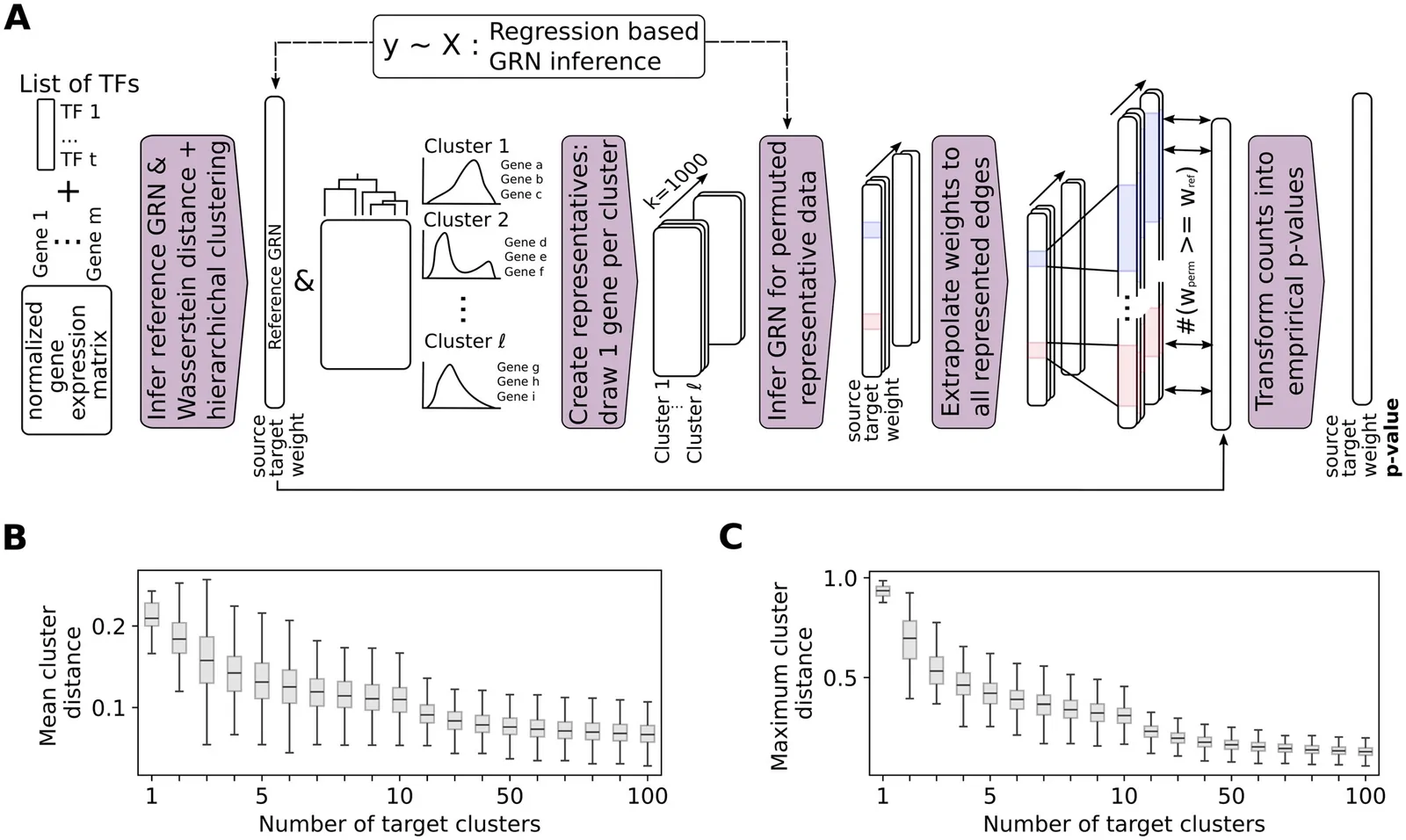
Method overview and assessment of distributional gene expression diversity in GTEx data. (A) As input, SignifiKANTE takes a gene expression matrix with columns scaled to unit variance (e.g. using *z*-score normalization) in combination with a regression-based GRN inference method of choice that infers a set of weighted TF-target edges from the data (reference GRN). Target genes are clustered using 1-Wasserstein distance-based hierarchical clustering, the regression-based GRN inference method is run k times with shuffled gene expression data for one representative per cluster with Dask-based parallelization over the target gene clusters, and the resulting edge weights between TFs and cluster representatives are used to compute approximate empirical *P*-values for all edges in the reference GRN. (B, C) Distributions of mean and maximum pairwise intra-cluster 1-Wasserstein distances resulting from 1-Wasserstein distance-based hierarchical clustering of gene expression profiles for 30 tissue gene expression datasets obtained from GTEx.

SignifiKANTE (Fig. 1A) exploits this observation as follows: We start by inferring a reference GRN from a column-normalized gene expression matrix and a list of TFs (candidate regulators) provided as input, using any regression-based GRN inference method. Moreover, we group all target genes based on the 1-Wasserstein distance into a constant number of ℓ clusters. For a user-specified number k of permutations (k determines the *P*-value resolution, default: k=1000), we then draw one representative gene per cluster (through random selection or by computing the cluster medoid), permute its expression vector, and infer TF-target edges from the shuffled target gene vector. The edge weights of the shuffled representative are extrapolated back onto all edges belonging to the respective cluster. We then count how often the edge weight inferred from the permuted data exceeds the corresponding reference edge weight, and turn this into an empirical *P*-value per edge. We thus obtain *P*-values for all edges in the reference GRN, which can optionally be adjusted for multiple testing, e.g. using Benjamini-Hochberg (BH) correction.

**Figure 2:**
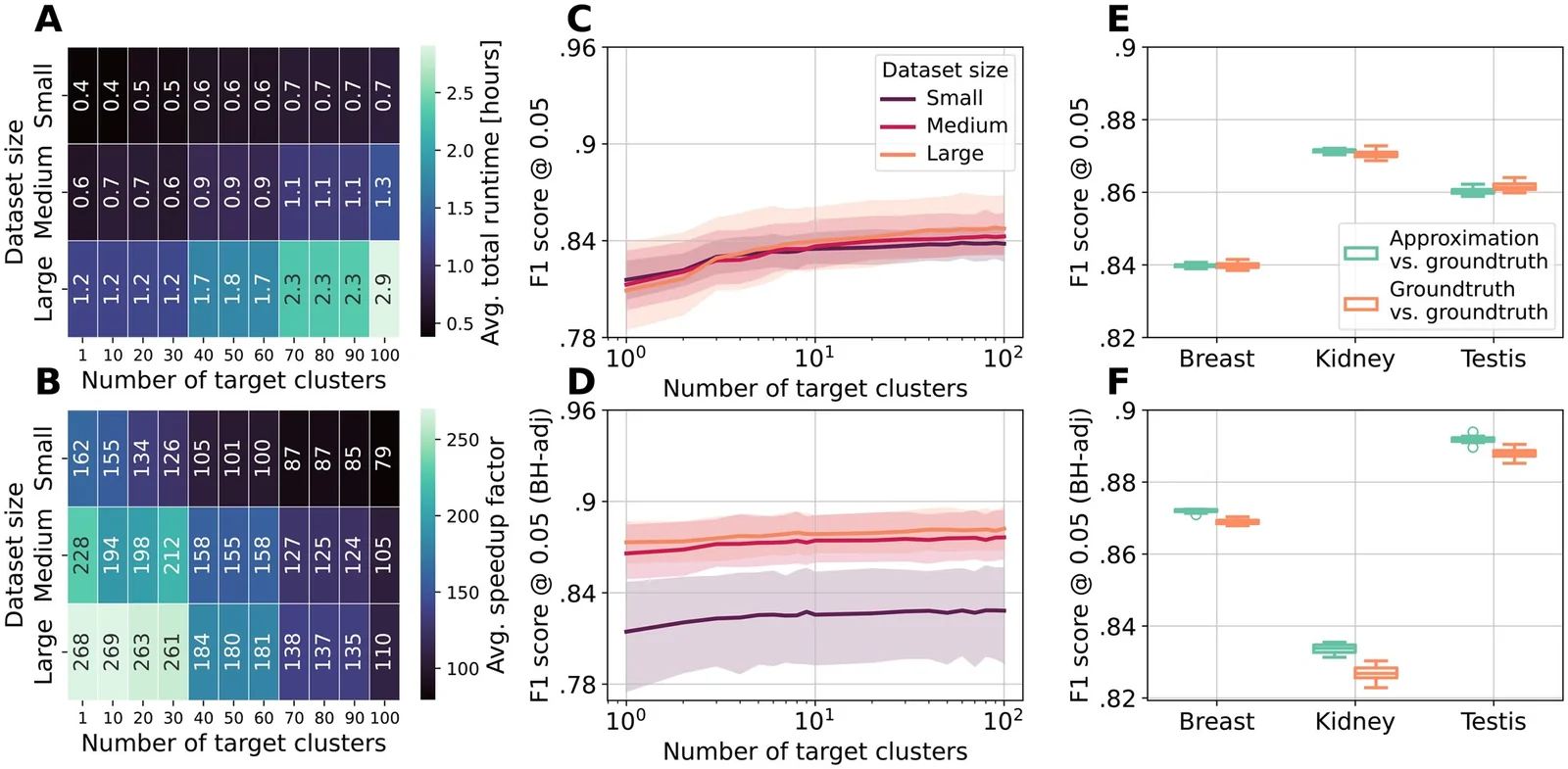
Comparison of SignifiKANTE (with GRNBoost2) to exact empirical *P*-value computation for GRNs as implemented in DIANE. (A) Mean SignifiKANTE runtimes across small (n<500), medium (500≤n≤1000), and large (n>1000) GTEx datasets for varying numbers of target clusters ℓ. (B) Corresponding mean speed-up factors in comparison to exact *P*-value computation. (C, D) F1 scores for varying ℓ obtained via comparison of edges in GRNs with significant exact and approximate *P*-values at 0.05 significance level, both without (C) and with (D) BH correction. In both cases, groundtruth positives are edges that have a significant exact *P*-value, and predicted positives are edges that have a significant approximate *P*-value. (E, F) F1 scores measuring approximation quality of SignifiKANTE’s *P*-values against 10 groundtruth *P*-values and of 10 groundtruth *P*-value runs against themselves for significance level 0.05, both without (E) and with (F) BH correction.

When analyzing the clustering structure resulting from the Wasserstein-based hierarchical clustering, we observed a common pattern across *z*-score normalized gene expression data of all 30 GTEx tissues (Fig. 1B and C). Both mean and maximum pairwise intra-cluster distances show a pronounced decrease for one up to 10 target gene clusters, with only moderate improvements from 20 up to 100 clusters. This shows that distributional diversity of column-normalized gene expression vectors is relatively small, suggesting that already 10 to 100 clusters should yield accurate approximate *P*-values. For scRNA-seq data from human PBMC samples, a very similar pattern emerges (Fig. B.1 in Appendix B, available as supplementary data at *Bioinformatics* online).

For all of the experimental results presented in the following sections, we ran SignifiKANTE with k=1000 permutation and GRNBoost2 as the core GRN inference method (results for other GRN inference methods are shown in Appendix B, available as supplementary data at *Bioinformatics* online, as detailed below). We selected GRNBoost2 as default because it is one of the most popular regression-based GRN inference methods and has been shown to yield a good tradeoff between runtime and accuracy in previous benchmarks (Pratapa *et al.* 2020). As input list of candidate regulators, we used the widely used list of human TFs from the Stein Aerts Lab.

### 2.2 SignifiKANTE reduces runtime by orders of magnitude and yields highly accurate approximate *P*-values

First, we assessed accuracy of SignifiKANTE’s approximate *P*-values in comparison to exact empirical *P*-values computed with the DIANE-like approach explained in the introduction and in Algorithm A.1 in Appendix A, available as supplementary data at *Bioinformatics* online. For this, we used 30 healthy tissue gene expression datasets from GTEx (dataset overview in Table B.1 in Appendix B, available as supplementary data at *Bioinformatics* online), ran GRNBoost2 to compute reference GRNs for all of them, and computed exact and approximate *P*-values, using k=1000 permutations for both exact and approximate *P*-values and a varying number 1≤ℓ≤100 of target gene clusters for the approximate *P*-values (note that the extremely parallelized computation of all exact *P*-values took two weeks, highlighting the practical infeasibility of the exact approach). We computed F1 scores between exact and approximate *P*-values by treating edges with exact empirical *P*-values below 0.05 (with and without BH correction) as groundtruth positives, and edges with significant approximate *P*-values (significance cutoff at *P* = 0.05) as predicted positives.

Figure 2 shows the results with random selection of cluster representatives (using medoids as cluster representatives performed slightly worse, see Fig. B.2 in Appendix B, available as supplementary data at *Bioinformatics* online), aggregated over tissue datasets with similar numbers of samples (n<500, 500≤n≤1000, and n>1000). SignifiKANTE was orders of magnitude faster than the exact DIANE-like *P*-value computation, reducing runtimes from days or weeks to hours. Even when using ℓ=100 target gene clusters, SignifiKANTE runtimes fall below three hours on average on large datasets and below one hour on smaller ones (Fig. 2A). When using ℓ=10 target gene clusters, average runtimes of little more than one hour can be achieved also for the large datasets. Depending on the dataset sizes and the number of target gene clusters, these runtimes correspond to average speedup factors between 79 on small datasets and 269 on larger ones (Fig. 2B), resulting in an average 80- to 400-fold reduction in estimated CO_2_ emission (Fig. B.2 in Appendix B, available as supplementary data at *Bioinformatics* online).

Regarding the accuracy of the approximate *P*-values (Fig. 2C and D), we observe that, already for ℓ=10 target gene clusters, F1 scores always lie above a minimal value of around 0.78, with a further improvement for increasing ℓ. Moreover, F1 scores tend to be higher on tissues with larger sample sizes, especially when applying BH correction (Fig. 2D). This also coincides with lower mean absolute errors between SignifiKANTE’s approximate and groundtruth *P*-values on tissues with larger sample sizes (Fig. B.2 in Appendix B, available as supplementary data at *Bioinformatics* online). To put the observed F1 scores in the context of the intrinsic stochasticity of permutation-based empirical *P*-values, we ran 10 independent groundtruth calculations for a subset of three tissues (since already one groundtruth computation took days to weeks, doing this for all 30 tissues was computationally infeasible). We then analyzed pairwise F1 scores among all pairwise combinations of the 10 groundtruths by treating one as the reference and the other one as the approximation (true-positive edges are hence those that appear in both the reference and the approximation while false positive edges appear only in the approximation). Figure 2E and F presents pairwise F1-scores of the 10 groundtruths against themselves (“Groundtruth versus groundtruth”) and against SignifiKANTE’s approximate *P*-values, using ℓ=100 target gene clusters (“Approximate versus groundtruth”). Our results demonstrate that, both with and without BH correction, the F1 scores achieved by SignifiKANTE match the F1 scores of the pairwise groundtruth comparisons. This shows that the approximation quality of SignifiKANTE’s *P*-values is already optimal up to variation in the computation of the exact permutation-based *P*-values and the inherent stochasticity of the GRN inference method GRNBoost2.

Results for GTEx data and GReNaDIne’s LASSO regression as core GRN inference method are shown in Fig. B.3 in Appendix B, available as supplementary data at *Bioinformatics* online. On a subset of the GTEx tissues, we additionally tested SignifiKANTE with GReNaDIne’s ridge regression (Fig. B.4, available as supplementary data at *Bioinformatics* online) and GENIE3 (Fig. B.5, available as supplementary data at *Bioinformatics* online) as core GRN inference method. Moreover, we evaluated SignifiKANTE with GRNBoost2 on three scRNA-seq datasets (Fig. B.6, available as supplementary data at *Bioinformatics* online). Results remain largely consistent with the findings presented here, showing that SignifiKANTE efficiently computes high-quality approximate empirical *P*-values for GRNs inferred from both bulk and single-cell gene expression data with different regression-based GRN inference methods.

**Figure 3:**
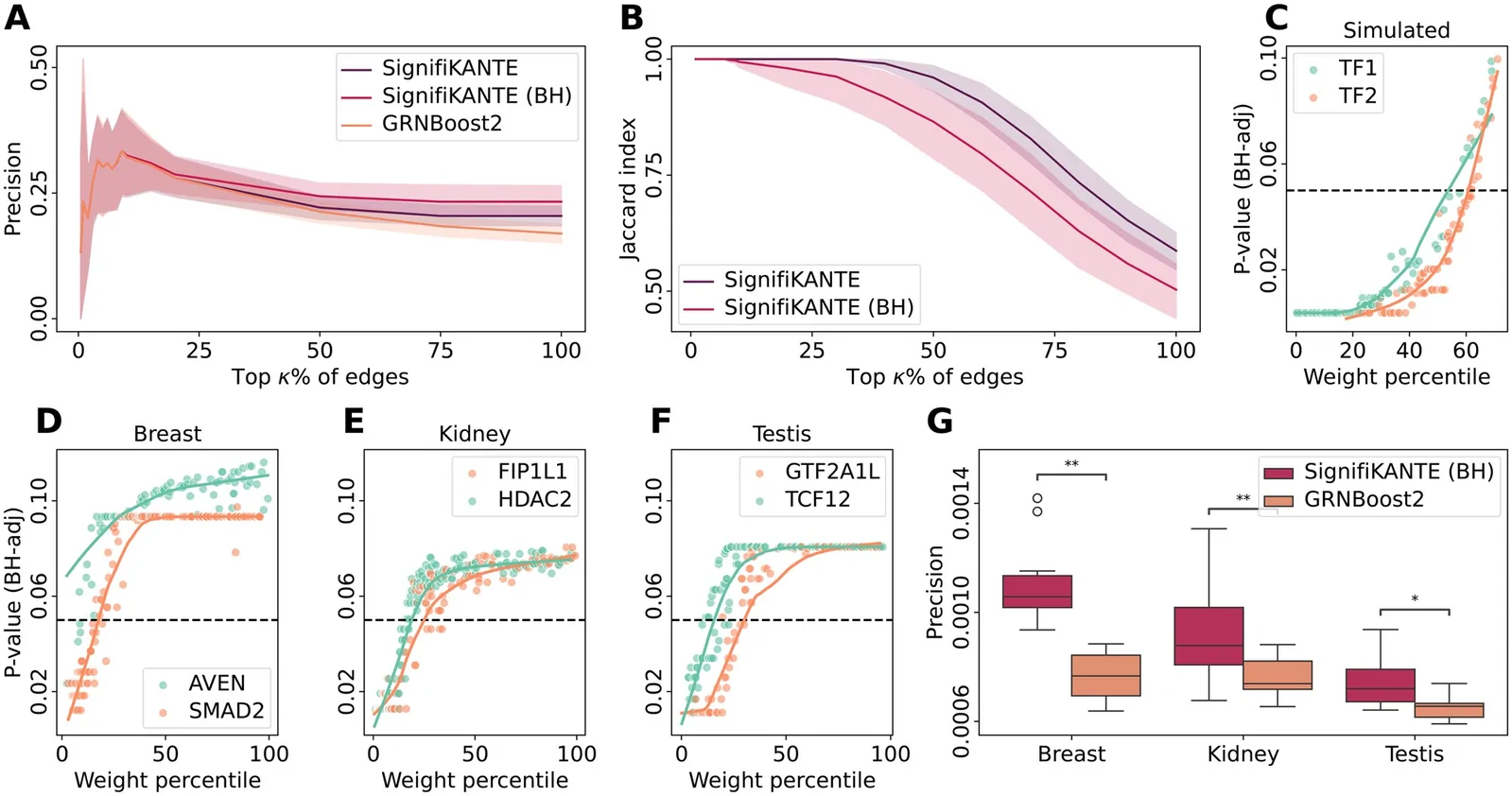
Effect of significance filtering with SignifiKANTE on GRNs inferred with GRNBoost2 from simulated and real-world data. (A) Precision of top κ% edges (sorted by weights) inferred from simulated expression data against groundtruth GRN used for simulation, using all GRNBoost2 edges, all significant edges of SignifiKANTE (significance cutoff at α=0.05, ℓ=10 target gene clusters), and all significant edges of SignifiKANTE with BH correction (FDR control at q=0.05, ℓ=10). The groundtruth GRN includes five TFs and at most 50 targets per TF. (B) Pairwise Jaccard indices between GRNBoost2 and SignfiKANTE edge sets for the edge cutoffs from (A). (C–F) Edge weight percentiles (low percentiles correspond to large weights) and BH-adjusted SignifiKANTE *P*-values for TF-target gene links of exemplary pairs of TFs on simulated data (C, FDR control at q=0.05, ℓ=10), and GTEx data for breast, kidney, and testis (D–F, FDR control at q=0.05, ℓ=100). Regression lines are fitted using locally weighted scatterplot smoothing. (G) Distributions of precision values between all GRNBoost2 edges inferred from breast, kidney, and testis GTEx data and only significant edges computed by SignifiKANTE (BH correction, and FDR control at q=0.05, ℓ=100). Known TF-target interactions from the CollecTRI database (Müller-Dott *et al*. 2023) were treated as groundtruth. Using different random seeds, we ran GRNBoost2 followed by SignifiKANTE ten times on the same data, and then assessed statistical significance via the Wilcoxon signed-rank test for matched samples.

**Figure 4:**
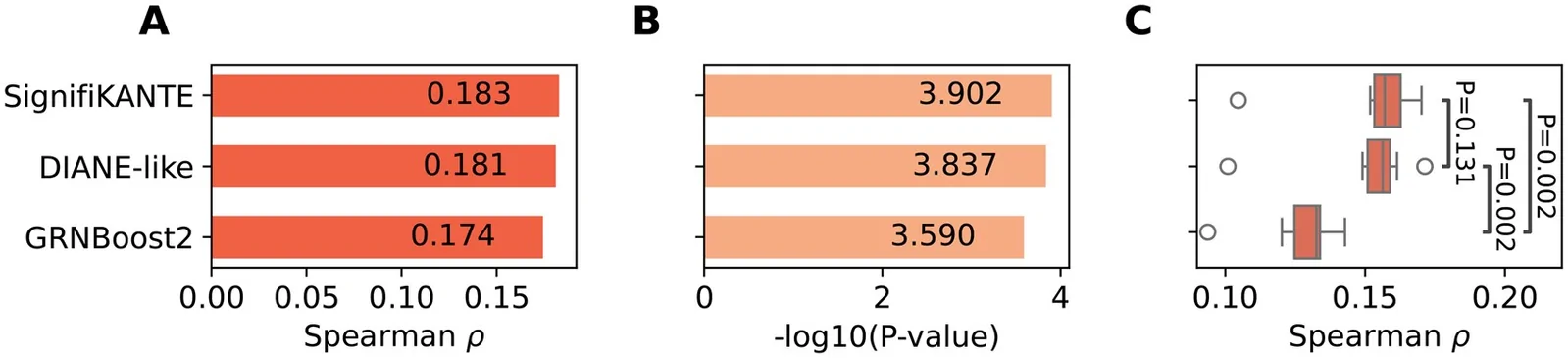
Comparison of edge similarity between GRNs inferred for different GTEx tissues with the BRENDA tissue ontology. (A) Spearman correlations of edge-wise Jaccard distances between GRNs and BTO-based shortest path distances across all 30 GTEx tissues, using GRNs containing, respectively, all GRNBoost2 edges and all significant edges for BH-corrected (FDR control at q=0.05) exact DIANE-like *P*-values and approximate *P*-values from SignifiKANTE (ℓ=100). (B) *P*-values corresponding to Spearman correlation coefficients shown in (A). (C) Distributions of Spearman correlations of BTO-based shortest path distances and edge-wise Jaccard distances between GRNs, with GRNs filtered to the κ most highly expressed target genes for κ∈{100,200,…,1000}. Pairwise *P*-values were computed with the two-sided Wilcoxon signed-rank test with pairing based on κ.

### 2.3 SignifiKANTE improves precision of inferred GRNs

Next, we asked to which extent SignifiKANTE improves upon the underlying GRN inference method GRNBoost2. For this, we simulated gene expression datasets in such a way that a given groundtruth GRN has a strong effect on the gene expression profiles, used vanilla GRNBoost2, and GRNBoost2 with SignifiKANTE (with and without BH correction) to infer GRNs from the simulated datasets, and then assessed the inferred GRNs’ precisions in comparison to the groundtruth GRNs underlying the simulation. Figure 3A and B shows the obtained precisions for the top κ% of edges with the largest weights in the different networks for varying κ, as well as pairwise Jaccard indices between the GRNBoost2 edge sets and the edge sets obtained with SignifiKANTE. The underlying groundtruth GRN consists of five TFs and 50 targets per TF, while precision values for two further groundtruth configurations are shown in Fig B.7 in Appendix B, available as supplementary data at *Bioinformatics* online. For edges with large weights, the edge sets for the filtered and unfiltered networks are close to identical and hence yield very similar precision values. This indicates that edges with a high weight are generally statistically stable and have a significant *P*-value, which is an expected result. However, in the lower ranking edges, SignifiKANTE improves the precision on the simulated data, by removing statistically weak edges—especially when used in combination with BH correction. These results suggest that SignifiKANTE’s significance filtering effectively separates relevant from non-relevant low-weight TF-target gene links. Moreover, we observed that the relation between edge weight percentiles and *P*-values is often shifted between sets of outgoing edges incident with different TFs (Fig. 3C). This implies that, with a purely weight-based GRN filtering strategy that does not make use of SignifiKANTE’s *P*-values, one either retains many non-relevant edges for TFs where the relation between weight percentiles and *P*-values is left-shifted (turquoise TF1 in Fig. 3C) or looses relevant ones for TFs where this relation is shifted to the right (orange TF1 in Fig. 3C).

In addition to our tests on simulated data, we evaluated the ability of SignifiKANTE to filter out irrelevant edges from the given reference GRNBoost2 GRN on the three GTEx tissues we also used for analysis of SiginifiKANTE’s approximate *P*-values in the context of the groundtruth variability. For this, we first filtered the respective reference GRNs for the most expressive regulatory interactions by subsetting it to edges targeting the 1000 most highly expressed target genes and then compared the GRNs of vanilla GRNBoost2 to GRNs filtered by SignifiKANTE (with BH correction, since this yielded the best results on simulated data). As for simulated data, we observe TF-specific shifts in the relation between edge weight percentiles and SignifiKANTE’s *P*-values (Fig. 3D–F). This shows that, also for real-world data, the commonly used GRN filtering strategy to consider only the top κ% of edges with the largest weights either leads to a systematic inclusion of spurious links for some TFs (turquoise TFs in Fig. 3D–F) or to a systematic exclusion of relevant links for other TFs (orange TFs in Fig. 3D–F). As an example, in Fig. 3D, most of SMAD2’s regulatory edges within the 25th weight percentile are significant (*P*-value below dashed line at 0.05), while AVEN’s regulatory edges at the same weight percentile are mostly not significant. This highlights that weight-based cutoffs are not necessarily comparable between different TFs. Furthermore, on the same set of GTEx tissues, we compared the enrichment of known TF-target interactions from the CollecTRI database (Müller-Dott *et al.* 2023) within the complete GRNBoost2 GRNs and SignifiKANTE’s filtered FDR-controlled GRNs (Fig. 3G). For all three tissues, the precision values computed when treating CollecTRI as groundtruth increase slightly but significantly compared to the vanilla GRNBoost2 GRNs. We also computed recall values corresponding to the precision values shown in Fig. 3A and G (Fig. B.7 in Appendix B, available as supplementary data at *Bioinformatics* online). However, since SignifiKANTE computes subnetworks of the GRNs inferred by GRNBoost2, we know *a priori* that recall is higher for GRNBoost2 than for the filtered networks, which is why precision is the more relevant metric for our study.

### 2.4 SignifiKANTE improves representation of the BRENDA tissue ontology

Finally, we analyzed how well SignifiKANTE’s FDR-controlled GRNs inferred for the 30 GTEx tissue datasets reflect known (dis-)similarities between tissues. For this, we computed GRN-based tissue distances for all 30 GTEx tissues and compared them to distances based on the BTO (Gremse *et al.* 2011), which we calculated by mapping the GTEx tissues to corresponding BTO terms (Table B.2 in Appendix B, available as supplementary data at *Bioinformatics* online) and then computing pairwise shortest path distances on the ontology graph. Pairwise GRN-based tissue distances were calculated as Jaccard distances of the edge sets, using the edge sets inferred by vanilla GRNBoost2 and the significant edges resulting from, respectively, exact DIANE-like *P*-values and approximate *P*-values computed with SignifiKANTE with 100 target gene clusters, both coupled with BH correction with FDR control at q=0.05.

Figure 4 shows how well the respective GRN-based tissue distances correlate with the BTO-based distances. Our analysis revealed that FDR-controlled GRNs from SignifiKANTE yield a higher Spearman rank correlation coefficient against BTO-based tissue distances than both DIANE-like FDR control and vanilla GRNBoost2 (Fig. 4A). The corresponding *P*-values are shown in Fig. 4B and indicate that all computed correlation coefficients are significant for α=0.05. To assess if the changes in correlation are significant, we subset the significance-filtered and unfiltered GRNs to the top κ most highly expressed target genes in the corresponding GTEx datasets for κ∈{100,200,…,1000} and then computed the Spearman correlation coefficients for the subnetworks induced by each κ. As shown in Fig. 4C, the significance-filtered GRNs yielded significantly larger correlation coefficients than the GRNs inferred by vanilla GRNBoost2; whereas there are no significant differences between filtering with SignifiKANTE and the exact DIANE-like approach. We can thus conclude that significance-based edge filtering with SignifiKANTE leads to GRNs that better reflect ontology-based tissue similarities than unfiltered GRNs inferred by vanilla GRNBoost2.

## 3 Discussion

In this article, we presented SignifiKANTE—a tool that allows to efficiently assess edge significance in GRNs inferred from realistically sized gene expression data. SignifiKANTE is not tied to a specific GRN inference method but can be run on top of every regression-based approach. Moreover, it does not require *ad hoc* filtering steps to decrease the computational burden. For this, SignifiKANTE computes approximate empirical *P*-values using clustering based on the 1-Wasserstein distance. In terms of quality, the approximate *P*-values match exact empirical *P*-values for GRN edges suggested in a previous study (Cassan *et al.* 2021), but can be computed in minutes to few hours instead of days to weeks. SignifiKANTE can be used on top of any regression-based GRN inference method; as default, we suggest the widely used method GRNBoost2. In comparison to GRNs inferred with vanilla GRNBoost2, SignifiKANTE’s GRNs show improved quality, especially in the tail of the TF-target gene links inferred by GRNBoost2 (for the edges with the highest weights, differences are small because most highly weighted edges are statistically significant). While the quality improvement is rather small in terms of magnitude, it is consistent across various metrics and datasets (precision in comparison to groundtruth and literature-curated TF-target gene links for simulated and real-world data, consistency with tissue ontology) and mostly statistically significant. In view of these results, we are convinced that SignifiKANTE is a valuable contribution to the network biology field and will help to make GRN inference from gene expression data more robust and reliable.

The main limitation of SignifiKANTE is that it is tied to regression-based GRN inference and cannot be used on top of other approaches such as the mutual information-based method ARACNe-AP (Lachmann *et al.* 2016). Another limitation is that SignifiKANTE’s Dask-based parallelization scheme over the target gene clusters induces a memory overhead that scales linearly with the number of used CPUs (Fig. B.6 in Appendix B, available as supplementary data at *Bioinformatics* online). Although memory requirements remained feasible even when using 30 CPUs (around 11 GiB on the three scRNA-seq datasets), there may be room for improvement of SignifiKANTE’s memory footprint by using alternative parallelization frameworks such as shared memory parallelization with OpenMP. We also observed that, when adjusting SignifiKANTE’s *P*-values for multiple testing via BH correction, in some instances no edges remain significant if the resolution of the uncorrected *P*-values is too small (Fig. B.8 in Appendix B, available as supplementary data at *Bioinformatics* online). If this happens, users can either increase the number of permutations k at the price of increased runtime or resort to a variant of Westfall-Young (WY) correction (Westfall and Young 1992) natively supported by SignifiKANTE, which requires fewer permutations than BH correction but controls the family-wise error rate (FWER) only at the level of individual target gene clusters and does not yield a global, GRN-wide multiple testing correction (details in Methods). Finally, our results for scRNA-seq data (Fig. B.6 in Appendix B, available as supplementary data at *Bioinformatics* online) show that SignifiKANTE is not only applicable to bulk data but can also handle the increased noise and dropout characteristic of scRNA-seq data. Since there are various tools for scRNA-seq data such as SCENIC (Aibar *et al.* 2017) and SwitchTFI (Martini *et al.* 2025) where regression-based GRN inference is used as a subroutine, a promising direction for future work is to explore using SignifiKANTE within these tools.

## 4 Materials and methods

### 4.1 Pseudocode of SignifiKANTE

A high-level algorithmic description of SignifiKANTE is shown in Algorithm 1. As input, SignifiKANTE requires a gene expression matrix X, a set of column indices T denoting TFs (candidate regulators), a regression-based GRN inference method GRN_inference() together with the matching aggregation operator ⊕ to aggregate TF-target gene links for different target genes, and two technical hyperparameters ℓ,k∈N. We start by normalizing the columns of the gene expression matrix to unit variance (Line 1, e.g. using *z*-score normalization), and then infer the GRN (E,w) for our data using GRN_inference() and ⊕ (Line 2). Next, we compute a matrix $D\in R^{m\times m}$ containing 1-Wasserstein distances for all pairs of columns in X (Line 3), and then use D to compute a gene clustering via agglomerative hierarchical clustering (Line 4). The number of gene clusters ℓ is a hyperparameter. Larger ℓ result in a more fine-grained representation of the original gene expression space but increase runtime.

We then compute approximate empirical *P*-values for all edges (t,j)∈E using a sped-up version of the approach presented in DIANE, parametrized with the user-specified number of permutations k. For each edge (t,j)∈E, we maintain a zero-initialized integer c(t,j) (Line 6) that counts how often weights obtained with GRN_inference() upon permutation of the target gene expression vector exceed the weight w(t,j) obtained with the real data. However, instead of computing a full randomized GRN for each of the k rounds of permutations as in DIANE, we only compute background weights for edges toward one representative $r_{l}$ per gene cluster $C_{l}$ in each iteration (Lines 10–12). For drawing $r_{l}$ (Line 8), we implemented two strategies: either using the cluster medoid (computed based on the 1-Wasserstein distance) or drawing a random cluster representative in each iteration. For the update of the edge counts c(t,j), the target gene j of a given edge (t,j)∈E from the reference GRN *G* is mapped to its corresponding cluster representatives $r_{l}$ and the edge count of c(t,j) is increased if the weight $\tilde{w}(t,r_{l})$ of the representative edge in the GRN obtained upon permutation of column $r_{l}$ is higher than the weight w(t,j) in the reference GRN G (Lines 15, 16).

**Algorithm 1** SignifiKANTE **Input:** Gene expression matrix $X\in {\mathbb{R}}^{n,m}$, set T⊆{1,…,m} of indices denoting TFs, regression-based GRN inference method GRN_inference: (X,y)↦(E,w) with E containing edges (t,j) with weights $w(t,j)\in R_{>0}$ from columns t of X to target gene j represented by expression vector y and operator ⊕ to aggregate the regulatory links over all target genes, number of permutations k, number of target gene clusters ℓ.

**Output 1:** Uncorrected approximate *P*-values $\tilde{p}(e)\in [0,1]$ for all TF-target gene edges e∈T×{1,…,m}.

**Output 2 (optional):** Cluster-level FWER-controlled approximate *P*-values $\hat{p}(e)\in [0,1]$ for all TF-target gene edges e∈T×{1,…,m} obtained via WY correction.

1: X←normalize_columns(X)

2: $\left(E,w)\leftarrow {⊕}_{i=1}^{m}\mathrm{GRN}\_\mathrm{inference}(X_{•,T\setminus \{i\}},X_{•,i}\right)$

3: D←compute_wasserstein_dists_for_all_column_pairs(X)

4: $\left\{C_{1},\ldots ,C_{\ell }\}\leftarrow \mathrm{agglomerative}\_\mathrm{clustering}(D,\ell \right)$

5: **for** each (t,j)∈E  **do** 

6:  c(t,j)←0

7:  $\hat{c}(t,j)\leftarrow 0$  ▹ only for WY correction

8: **for**  l=1,…,ℓ  **do**  ▹ parallelized with Dask

9:  **for**  i=1,…,k  **do** 

10:   $r_{l}\leftarrow \mathrm{draw}\_\mathrm{cluster}\_\mathrm{representative}(C_{l})$

11:   ${\tilde{y}}_{r_{l}}\leftarrow \mathrm{shuffle}(X_{•,r_{l}})$

12:   $\left(\tilde{E},\tilde{w})\leftarrow \mathrm{GRN}\_\mathrm{inference}(X_{•,T\setminus \{r_{l}\}},{\tilde{y}}_{r_{l}}\right)$

13:   ${\tilde{w}}_{l}={\text{max}}_{t\in T}\tilde{w}(t,r_{l})$  ▹ only for WY correction

14:   **for** each (t,j)∈E  **do** 

15:    **if**  $j\in C_{l}\wedge \tilde{w}(t,r_{l})\geq w(t,j)$  **then** 

16:     c(t,j)←c(t,j)+1

17:    **if**  $j\in C_{l}\wedge {\tilde{w}}_{l}\geq w(t,j)$  **then** 

18:     $\hat{c}(t,j)\leftarrow \hat{c}(t,j)+1$  ▹ only for WY correction

19: **for** each (t,j)∈E  **do** 

20:  $\tilde{p}(t,j)\leftarrow (c(t,j)+1)/(k+1)$  ▹ uncorrected *P*-values

21:  $\hat{p}(t,j)\leftarrow (\hat{c}(t,j)+1)/(k+1)$  ▹ WY-corrected *P*-values

22: **return**  $\tilde{p}$, $\hat{p}$

Upon computing k randomized GRNs for representatives of all ℓ target gene clusters, SignifiKANTE computes approximate empirical *P*-values for all edges (t,j)∈E in the GRN inferred from the original data (Line 20). These *P*-values can optionally be adjusted for multiple testing (with the number of tests set to $\left|E\right|$) using BH correction, which is natively supported in the SignifiKANTE Python package. Note that FDR cutoffs on the adjusted *P*-values should be chosen in light of the resolution 1/(k+1) of the empirical *P*-values. For instance, with SignifKANTE’s default k=1000, the smallest possible non-adjusted empirical *P-*value is just below 0.001, and in preliminary experiments we thus obtained mostly empty result sets when controlling the FDR at q≤0.01. This motivated us to select q=0.05 as default.

However, even with this setup, the power of SignifiKANTE in combination with BH correction remains limited due to the large number of tested hypotheses. As an alternative multiple testing correction strategy, SignifiKANTE therefore incorporates a variant of WY correction that allows for FWER control among edges $e\in T\times C_{l}$, individually for all target gene clusters $C_{l}$ (the corresponding steps are marked by comments in Algorithm 1). Since WY correction directly yields multiple testing adjusted *P*-values with resolution 1/(k+1), it is better powered than BH correction, where raw *P*-values with resolution 1/(k+1) are corrected *post hoc*. However, our WY correction variant only controls for multiple testing within individual target gene clusters and thus cannot be used for the DIANE-like groundtruth approach where no target gene clusters are available. To enable a fair comparison, we therefore used BH correction for most of the results reported in this article.

### 4.2 Choice of the hyperparameters *ℓ* and *k*, the multiple testing correction strategy, and the clustering method

In order to provide the user of SignifiKANTE with a reasonable choice of the number of clusters ℓ on any given dataset, the SignifiKANTE package includes the heuristic function find_optimal_number_of_clusters. Given any normalized gene expression matrix, it computes the corresponding Wasserstein distance matrix once and iterates over all cluster number candidates ℓ=1,…,100. In each iteration, it computes the mean of all resulting cluster diameters, and terminates once the decrease in the mean diameter falls below 1.5% of the diameter of the whole Wasserstein distance matrix. Like this, we ensure that ℓ is small enough to let the user benefit from proper runtime speed-ups, but large enough such that the clusters properly cover the gene count distribution space. The suggested values of ℓ range from six to fourteen across all analyzed datasets with a median of ℓ=9 and a standard deviation of 1.85 (Fig. B.9 in Appendix B, available as supplementary data at *Bioinformatics* online).

Regarding the number of permutations k, we used k=1000 for most of our analyses. To nonetheless analyze the effect of k, we carried out an ablation study using both scRNA-seq and GTEx data and different core GRN inference methods (Fig. B.8 in Appendix B, available as supplementary data at *Bioinformatics* online): While faithfulness of approximate *P*-values with respect to exact DIANE-like *P*-values remains very stable across different k, k strongly affects the number of significant edges after BH correction on the scRNA-seq datasets, likely due to the *P*-value resolution limit of 1/(k+1). WY correction is very stable with respect to the choice of k and consistently leads to a higher number of significant edges than BH correction. We thus recommend using k=1000 as a default and resorting to WY correction if BH correction yields empty results.

We also analyzed the effect of using other clustering methods than agglomerative hierarchical clustering to obtain the target gene clusters $C_{l}$ in Line 4 of Algorithm 1. For this, we ran SignifiKANTE with spectral clustering and *K*-medoids clustering on the Wasserstein distance matrix D on three of the GTEx datasets (Fig. B.10 in Appendix B, available as supplementary data at *Bioinformatics* online). We obtained near-identical results with all clustering algorithms, showing that SignifiKANTE is robust with respect to the choice of this subroutine.

### 4.3 Implementation

For computing the matrix $D\in R_{\geq 0}^{m\times m}$ of pairwise 1-Wasserstein distances in Line 3 of Algorithm 1, we implemented a more efficient equivalent of SciPy’s wasserstein_distance() function, which allows us to handle large expression matrices with several thousand gene columns by relying on parallelization over all pairs of genes and code compilation to C using the Python just-in-time compiler Numba. As a first step, our implementation sorts all gene columns separately, which has a runtime complexity of O(mn log n). Subsequently, we iterate over all pairs of genes, merge the already sorted column vectors, and compute discrete CDFs. This second step is dominated by the merging operation using numpy.searchsorted with a runtime complexity of O(n log n), which yields a total runtime complexity of $O(m^{2}n\;\text{log}\;n)$. Memory requirements are dominated by the space needed to keep the distance matrix D in memory, leading to a space complexity of $O(m^{2})$. We benchmarked the computational requirements of computing D (Fig. B.11 in Appendix B, available as supplementary data at *Bioinformatics* online) and found that, in the bulk data regime (few samples: n=1000, high number of genes: m=20 000), wall-clock time and peak memory usage remained below 30 minutes and 6.5 GiB, respectively. In the single-cell regime (many samples: n=10 000, highly variable genes only: m=2000), computation required less than 4 minutes and 601 MiB of peak memory. Agglomerative hierarchical clustering based on D (Line 4 in Algorithm 1) is implemented using sklearn’s cluster. AgglomerativeClustering class to obtain ℓ target gene clusters; for *K*-medoids and spectral clustering (which can be selected as alternative clustering methods by the user), we used sklearn_extra.cluster.KMedoids and sklearn.cluster.SpectralClustering. Note that by setting ℓ=m, the user can also use SignifiKANTE to compute exact empirical *P*-values as in DIANE.

Since GRNBoost2 in its Arboreto implementation is one of the most popular GRN inference tools, we decided to implement SignifiKANTE as an add-on to Arboreto. Arboreto uses Dask for parallelizing the main loop over the target genes in regression-based GRN inference (i.e. the regression models $y_{j}∼f_{j}(X_{•,T\setminus \{j\}})$ are fit in independent Dask processes). For SignifiKANTE, we changed the logic of the parallelization to avoid the repeated costly creation and shutdown of Dask processes in each permutation. Instead of making a full call to Arboreto’s GRN inference function for each permutation, we parallelize the loop over the target gene clusters (Line 8 in Algorithm 1) and, within each Dask process, compute the empirical *P*-values for the partial GRN containing edges toward target genes from the respective cluster. SignifiKANTE provides out-of-the-box support for the two GRN inference methods GRNBoost2 and GENIE3 implemented in Arboreto, as well as for GReNaDIne’s LASSO-based approach. The latter implementation also serves as a template which showcases how users can easily extend SignifiKANTE with any regression-based GRN method.

### 4.4 Real-world and simulated gene expression data

We used both real-world and simulated gene expression data for our study. Real-world bulk RNA-sequencing data for 30 healthy tissues was obtained in transcripts per million (TPM) from GTEx and was used with minimal processing (column-wise *z*-score normalization, genes expressed in fewer than 10% of samples filtered out, numbers of samples and genes after preprocessing are shown in Table B.1 in Appendix B, available as supplementary data at *Bioinformatics* online). Real-world scRNA-seq datasets of dendritic cells, CD8-positive T cells, and naïve killer cells from human PBMC samples were obtained from De Simone *et al.* (2025) and contain 405, 613, 247 cells, respectively with 1176 genes selected after stringent filtering (preprocessing steps are detailed in Appendix A, available as supplementary data at *Bioinformatics* online, dataset sizes in Table A.1 in Appendix A, available as supplementary data at *Bioinformatics* online). The simulated data underlying our comparison of GRNBoost2 with and without edge filtering based on SignifiKANTE’s *P*-values was generated with the scMultiSim package (Li *et al.* 2025), which allows to simulate gene expression data from an underlying groundtruth GRN. We used subnetworks of the CollecTRI GRNs as a base for our simulation, randomly subsampled to s TFs and min{t,deg(i)} unique target genes per randomly sampled TF i (deg(i) is the outdegree of the TF i in CollecTRI), with (s,t)∈{(5,50),(10,40),(20,30)}. Using scMultisim, and setting a strong induced effect for these subnetworks, we then simulated 10 gene expression datasets with n=500 cells for each (s,t) configuration, containing m∈{82,159,286} genes on average.

### 4.5 Evaluation metrics for comparison against exact DIANE-like *P*-values

For each edge e∈E in the reference GRN inferred from a GTEx dataset X, we computed exact *P*-values p(e) with the DIANE-like approach (Algorithm A.1 in Appendix A, available as supplementary data at *Bioinformatics* online) and approximate *P*-values $\tilde{p}(e)$ with SignifiKANTE. Due to the immense computational cost of the exact *P*-value computation task and a job time limit of 24 hours on the HPC systems provided by the Erlangen National High Performance Computing Center (NHR@FAU), we chunked the list of target genes into several batches for each tissue. For this, we set up a Slurm array job, which could then iterate over all tissues’ target gene batches and compute exact *P*-values on the respective chunk in under 24 hours, using Dask parallelization on 30 CPU cores. Like this, we were able to compute exact *P*-values for all 30 GTEx datasets on our HPC environment. Runtimes were recorded using Python’s time module. To compute the runtimes per tissue for the exact *P*-value computation that exceeded our 24 hours job time limit, we summed up the runtimes of the corresponding target gene batches. CO_2_ emission of exact and approximate *P*-value computation was estimated based on the OfflineEmissionsTracker module from the CodeCarbon Python package (Courty *et al.* 2024).

Given the exact and approximate *P*-values, we computed multiple testing-adjusted *P*-values $p_{\text{BH}}(e)$ and ${\tilde{p}}_{\text{BH}}(e)$, using BH correction. Then, we constructed four edge sets $E^{⋆}=\{e\in E|p(e)<0.05\}$, ${\tilde{E}}^{⋆}=\{e\in E|\tilde{p}(e)<0.05\}$, $E_{\text{BH}}^{⋆}=\{e\in E|p_{\text{BH}}(e)<0.05\}$, and ${\tilde{E}}_{\text{BH}}^{⋆}=\{e\in E|{\tilde{p}}_{\text{BH}}(e)<0.05\}$, and computed F1 scores treating $E^{⋆}$ as groundtruth for ${\tilde{E}}^{⋆}$ and $E_{\text{BH}}^{⋆}$ as groundtruth for ${\tilde{E}}_{\text{BH}}^{⋆}$. For the “Groundtruth versus groundtruth” and “Approximation versus groundtruth” comparisons (Fig. 2E and F), we computed a single reference GRN with edge set E and used it as input for 10 runs of the exact empirical *P*-value computation with different random seeds, as well as for one run of the approximate empirical *P*-value computation. By using the same reference GRN for all runs, only the inherent stochasticity of permutation-based *P*-value computation is reflected in the resulting groundtruth *P*-values ${\left(p^{i}(e)\right)}_{i=1}^{10}$, ${\left(p_{\text{BH}}^{i}(e)\right)}_{i=1}^{10}$ and approximate *P*-values $\tilde{p}(e)$, ${\tilde{p}}_{\text{BH}}(e)$ for e∈E. Thresholding these *P*-values as described above yields edge sets ${\left(E^{⋆}\right)}_{i=1}^{10}$, ${\left(E_{\text{BH}}^{⋆}\right)}_{i=1}^{10}$ and ${\tilde{E}}^{⋆}$, ${\tilde{E}}_{\text{BH}}^{⋆}$. We computed distributions of groundtruth-versus-groundtruth F1 scores by comparing $E^{⋆,i}$ against $E^{⋆,j}$ and $E_{\text{BH}}^{⋆,i}$ against $E_{\text{BH}}^{⋆,j}$ for i≠j, and approximation-versus-groundtruth F1 scores by comparing $E^{⋆,i}$ against ${\tilde{E}}^{⋆}$ and $E_{\text{BH}}^{⋆,i}$ against ${\tilde{E}}_{\text{BH}}^{⋆}$ for all i.

### 4.6 Evaluation metrics for comparison against vanilla GRNBoost2

For the comparison on the simulated data, we constructed edge sets $E^{\kappa }$ as the sets of top κ% of edges (sorted by edge weights) of the edge set E inferred by vanilla GRNBoost2, as well as edge sets ${\tilde{E}}^{⋆,\kappa }=E^{\kappa }\cap {\tilde{E}}^{⋆}$ and ${\tilde{E}}_{\text{BH}}^{⋆,\kappa }=E^{\kappa }\cap {\tilde{E}}_{\text{BH}}^{⋆}$ (${\tilde{E}}^{⋆}$ and ${\tilde{E}}_{\text{BH}}^{⋆}$ defined as above). For $F^{\kappa }\in \{E^{\kappa },{\tilde{E}}^{⋆,\kappa },{\tilde{E}}_{\text{BH}}^{⋆,\kappa }\}$, we then computed precision and recall at κ as $\text{precision}(F^{\kappa })=|F^{\kappa }\cap E_{\text{GT}}|/|F^{\kappa }|$ and $\text{recall}(F^{\kappa })=|F^{\kappa }\cap E_{\text{GT}}|/|E_{\text{GT}}|$, where $E_{\text{GT}}$ is the groundtruth network from the data simulation. We also computed pairwise Jaccard indices $JI_{\kappa }(E,{\tilde{E}}^{⋆})=|E^{\kappa }\cap {\tilde{E}}^{⋆,\kappa }|/|E^{\kappa }\cup {\tilde{E}}^{⋆,\kappa }|$ and $JI_{\kappa }(E,{\tilde{E}}_{\text{BH}}^{⋆})=|E^{\kappa }\cap {\tilde{E}}_{\text{BH}}^{⋆,\kappa }|/|E^{\kappa }\cup {\tilde{E}}_{\text{BH}\;}^{⋆,\kappa }|$. Note that $E^{\kappa }⊇{\tilde{E}}^{⋆,\kappa }⊇{\tilde{E}}_{\text{BH}}^{⋆,\kappa }$ always holds, which implies $\text{recall}(E^{\kappa })\geq \text{recall}({\tilde{E}}^{⋆,\kappa })\geq \text{recall}({\tilde{E}}_{\text{BH}}^{⋆,\kappa })$. For the precision, there is no such *a priori* known monotonous relationship, which makes it the more relevant metric. For the GTEx data, we computed $\text{precision}(F)=|F\cap E_{\text{CollecTRI}}|/|F|$ and $\text{recall}(F)=|F\cap E_{\text{CollecTRI}}|/|E_{\text{CollecTRI}}|$ for $F\in \{E,E_{\text{BH}}^{⋆},{\tilde{E}}_{\text{BH}}^{⋆}\}$, where E and ${\tilde{E}}_{\text{BH}}^{⋆}$ are defined as before, $E_{\text{BH}}^{⋆}$ is the corresponding FDR-controlled GRN based on exact DIANE-like *P*-values, and $E_{\text{CollecTRI}}$ is a set of curated TF-target gene edges obtained from CollecTRI, which we used as reference. Due to the incompleteness and non-specificity of CollecTRI, $E_{\text{CollecTRI}}$ cannot be seen as a real gold-standard. However, it can be used to evaluate if the biological signal is improved.

To assess the correlation of pairwise edge-based Jaccard distances of GRNs with ontology-based tissue distances, we first conducted a manual mapping of the 30 GTEx tissue types onto BTO terms (see Table B.2 in Appendix B, available as supplementary data at *Bioinformatics* online). The graph representing the BTO was read into a NetworkX (Hagberg *et al.* 2008) graph object. Using NetworkX, we then computed pairwise shortest path distances between the mapped BTO terms in order to obtain our ontology-based distance matrix of tissues, against which we then compared our GRN-based distance matrices.

### 4.7 Computing environment used for benchmarking

All benchmarks have been conducted on the HPC resources provided by NHR@FAU. More specifically, we ran our benchmarks on the CPU-based compute cluster “Woody”, which is equipped with one Intel Xeon E3-1240 v5 (“Skylake”) CPU, one Intel Xeon E3-1240 v6 (“Kaby Lake”) CPU, and two Intel Xeon Gold 6326 (“Ice Lake”) CPUs. In all our benchmarks, we used a total of 30 cores for the parallelization with Dask.

## Supplementary Material

btag531_Supplementary_Data

## Data Availability

We deposited the FDR-controlled GRNs for all thirty GTEx tissues at https://doi.org/10.5281/zenodo.17581025. The list of human TFs was taken from https://resources.aertslab.org/cistarget/tf_lists/, GTEx data from https://www.gtexportal.org/home/downloads/adult-gtex/bulk_tissue_expression, and scRNA-seq data from SRA under accessions SRR28867545, SRR28867561, SRR28867562, and SRR28867563. CollecTRI was accessed using the decoupler package (Badia-i Mompel *et al.* 2022), and the BTO from https://www.brenda-enzymes.org/ontology.php?ontology_id=3.

## References

[btag531-B1] Aibar S , González-Blas CB, Moerman T et al SCENIC: single-cell regulatory network inference and clustering. Nat Methods 2017;14:1083–6. 10.1038/nmeth.4463 28991892 PMC5937676

[btag531-B2] Badia-i Mompel P , Vélez Santiago J, Braunger J et al decoupleR: ensemble of computational methods to infer biological activities from omics data. Bioinf Adv 2022;2:vbac016. 10.1093/bioadv/vbac016 PMC9710656 36699385

[btag531-B3] Cassan O , Lèbre S, Martin A. Inferring and analyzing gene regulatory networks from multi-factorial expression data: a complete and interactive suite. BMC Genomics 2021;22:387. 10.1186/s12864-021-07659-2 34039282 PMC8152307

[btag531-B4] Courty B et al mlco2/codecarbon: v2.4.1 (Version v2.4.1). Zenodo 2024. 10.5281/zenodo.11171501

[btag531-B5] De Simone M , Hoover J, Lau J et al A comprehensive analysis framework for evaluating commercial single-cell RNA sequencing technologies. Nucleic Acids Res 2025;53:gkae1186. 10.1093/nar/gkae1186 39675380 PMC11754665

[btag531-B6] Faith JJ , Hayete B, Thaden JT et al Large-scale mapping and validation of *Escherichia coli* transcriptional regulation from a compendium of expression profiles. PLoS Biol 2007;5:e8. 10.1371/journal.pbio.0050008 17214507 PMC1764438

[btag531-B7] Gremse M , Chang A, Schomburg I et al The BRENDA tissue ontology (BTO): the first all-integrating ontology of all organisms for enzyme sources. Nucleic Acids Res 2011;39:D507–13. 10.1093/nar/gkq968 21030441 PMC3013802

[btag531-B8] GTEx Consortium. The genotype-tissue expression (GTEx) project. Nat Genet 2013;45:580–5. 10.1038/ng.2653 23715323 PMC4010069

[btag531-B9] Hagberg AA , Schult DA, Swart PJ. Exploring network structure, dynamics, and function using NetworkX. In: Varoquaux G, Vaught T, Millman J (Eds.), Proceedings of the 7th Python in Science conference (SciPy 2008), 2008, pp. 11–15

[btag531-B10] Haury A-C , Mordelet F, Vera-Licona P et al TIGRESS: Trustful inference of gene REgulation using stability selection. BMC Syst Biol 2012;6:145. 10.1186/1752-0509-6-145 23173819 PMC3598250

[btag531-B11] Huynh-Thu VA , Irrthum A, Wehenkel L et al Inferring regulatory networks from expression data using tree-based methods. PLoS One 2010;5:e12776. 10.1371/journal.pone.0012776 20927193 PMC2946910

[btag531-B12] Ketteler A , Blumenthal DB. Demographic confounders distort inference of gene regulatory and gene co-expression networks in cancer. Brief Bioinform 2023;24:bbad413. 10.1093/bib/bbad413 37985453

[btag531-B13] Lachmann A , Giorgi FM, Lopez G et al ARACNe-AP: gene network reverse engineering through adaptive partitioning inference of mutual information. Bioinformatics 2016;32:2233–5. 10.1093/bioinformatics/btw216 27153652 PMC4937200

[btag531-B14] Lambert SA , Jolma A, Campitelli LF et al The human transcription factors. Cell 2018;172:650–65. 10.1016/j.cell.2018.01.029 29425488 PMC12908702

[btag531-B15] Li H , Zhang Z, Squires M et al scMultiSim: simulation of single-cell multi-omics and spatial data guided by gene regulatory networks and cell-cell interactions. Nat Methods 2025;22:982–93. 10.1038/s41592-025-02651-0 40247122 PMC12226025

[btag531-B16] Margolin AA , Nemenman I, Basso K et al ARACNE: an algorithm for the reconstruction of gene regulatory networks in a mammalian cellular context. BMC Bioinformatics 2006;7:S7. 10.1186/1471-2105-7-S1-S7 PMC1810318 16723010

[btag531-B17] Martini P , Hartebrodt A, de Almeida GP et al SwitchTFI: identifying transcription factors driving cell differentiation. Genome Biol 2025;26:410. 10.1186/s13059-025-03876-0 41331466 PMC12673684

[btag531-B18] Moerman T , Aibar Santos S, Bravo González-Blas C et al GRNBoost2 and Arboreto: efficient and scalable inference of gene regulatory networks. Bioinformatics 2019;35:2159–61. 10.1093/bioinformatics/bty916 30445495

[btag531-B19] Morgan D , Tjärnberg A, Nordling TEM et al A generalized framework for controlling FDR in gene regulatory network inference. Bioinformatics 2019;35:1026–32. 10.1093/bioinformatics/bty764 30169550

[btag531-B20] Müller-Dott S , Tsirvouli E, Vazquez M et al Expanding the coverage of regulons from high-confidence prior knowledge for accurate estimation of transcription factor activities. Nucleic Acids Res 2023;51:10934–49. 10.1093/nar/gkad841 37843125 PMC10639077

[btag531-B21] Pratapa A , Jalihal AP, Law JN et al Benchmarking algorithms for gene regulatory network inference from single-cell transcriptomic data. Nat Methods 2020;17:147–54. 10.1038/s41592-019-0690-6 31907445 PMC7098173

[btag531-B22] Schmitt P , Sorin B, Frouté T et al GReNaDIne: a data-driven python library to infer gene regulatory networks from gene expression data. Genes (Basel) 2023;14:269. 10.3390/genes14020269 36833196 PMC9957546

[btag531-B23] Tjärnberg A , Nordling TEM, Studham M et al Avoiding pitfalls in L1-regularised inference of gene networks. Mol Biosyst 2015;11:287–96. 10.1039/c4mb00419a 25377664

[btag531-B24] Wang C , Liu Z-P. Network activity evaluation reveals significant gene regulatory architectures during SARS-CoV-2 viral infection from dynamic scRNA-seq data. IEEE J Biomed Health Inform 2024;28:2373–84. 10.1109/JBHI.2024.3360529 38294926

[btag531-B25] Wang H , Qiu Y, Guo H et al Information-incorporated gene network construction with FDR control. Bioinformatics 2024;40:btae125. 10.1093/bioinformatics/btae125 38430463 PMC10937901

[btag531-B26] Westfall PH , Young SS. Resampling-Based Multiple Testing: Examples and Methods for p-Value Adjustment. Wiley Series in Probability and Statistics. Nashville, TN: John Wiley & Sons, 1992.

[btag531-B27] Yeh H-Y , Cheng S-W, Lin Y-C et al Identifying significant genetic regulatory networks in the prostate cancer from microarray data based on transcription factor analysis and conditional independency. BMC Med Genomics 2009;2:70. 10.1186/1755-8794-2-70 20025723 PMC2805685

