# Supplementary material for: SignifiKANTE: efficient P-value computation for gene regulatory networks
Source: Bioinformatics. 2026 Jul 22;42(9):btag531. doi: 10.1093/bioinformatics/btag531 (PMC13549948; doi:10.1093/bioinformatics/btag531)
Supplement: Supplementary file 1 [file btag531_supplementary_data.pdf]

SignifiKANTE: Efficient *P*-value computation for gene regulatory networks

Appendices

Fabian Woller[1,†], Paul Martini[1,†], Souptik Sen[1,2], David B. Blumenthal[1], and Anne Hartebrodt[1]

[1] Biomedical Network Science Lab, Friedrich-Alexander-Universität Erlangen-Nürnberg, Erlangen, Germany
{fabian.woller,paul.martini,david.b.blumenthal,anne.hartebrodt}@fau.de
[2] Peter L. Reichertz Institut für Medizinische Informatik, Medizinische Hochschule Hannover, Germany
Sen.Souptik@mh-hannover.de

A Supplementary methods

A.1 Exact empirical *P*-value computation for regression-based GRN inference

Algorithm A.1 shows how to compute empirical P-values for GRNs inferred from gene expression data using regression-based approaches. The algorithm generalizes the approach that is implemented for GENIE3 [6] in the DIANE dashboard for GRN inference and analysis [1] to arbitrary regression-based GRN inference methods. Note that the two filtering steps used in DIANE prior to P-value computation (restricting GRN inference to differentially expressed genes and applying a hard threshold on the weights $w(t,j)$ to a achieve a small edge density) are not reflected in Algorithm A.1.

Algorithm A.1: DIANE-like empirical P-value computation for regression-based GRN inference

Input: Gene expression matrix $X \in \mathbb{R}^{n,m}$, set $T \subseteq \{1,\ldots,m\}$ of indices denoting TFs, regression-based GRN inference method `GRN_inference` : $(X, y) \mapsto (E, w)$ with E containing edges (t,j) with weights $w(t,j) \in \mathbb{R}_{>0}$ from columns t of X to target gene j represented by expression vector y and operator $\bigoplus$ to aggregate the regulatory links over all target genes, number of permutations k.

Output: TF-target gene edges $e \in T \times \{1,\ldots,m\}$ with empirical P-values $p(e) \in [0,1]$.

$X \leftarrow$ `normalize_columns`(X);
$(E, w) \leftarrow \bigoplus_{j=1}^{m}$ `GRN_inference`$(X_{\bullet,T\setminus\{j\}}, X_{\bullet,j})$;
for $(t,j) \in E$ **do** $c(t,j) \leftarrow 0$;
for $j = 1,\ldots,m$ **do**
    for $i = 1,\ldots,k$ **do**
        $\widetilde{y}_j \leftarrow$ `shuffle`$(X_{\bullet,j})$;
        $(\widetilde{E}, \widetilde{w}) \leftarrow$ `GRN_inference`$(X_{\bullet,T\setminus\{j\}}, \widetilde{y}_j)$;
        for $(t,j) \in E$ **do**
            if $\widetilde{w}(t,j) \geq w(t,j)$ **then**
                $c(t,j) \leftarrow c(t,j) + 1$;
for $(t,j) \in E$ **do** $p(t,j) \leftarrow (c(t,j)+1)\,/\,(k+1)$;
return E, p;

A.2 Application of SignifiKANTE to single-cell RNA sequencing data

The three single-cell RNA sequencing (scRNA-seq) datasets of dendritic cells, natural killer cells, and CD8+ T cells [2] were preprocessed using scanpy with strict filtering criteria. Cells with fewer than 500 detected

† These authors contributed equally to this work.

genes, cells for which mitochondrial transcripts accounted for more than 20% of total expression, and genes expressed in fewer than 50 cells or less than 5% of all cells were filtered out. Putative doublets were identified and removed using the `scanpy.pp.scrublet()` function. Following quality control, gene expression counts were normalized by total counts per cell and subsequently transformed using the $\log(1 + x)$ function to stabilize variance. Among the genes identified as highly variable (`scanpy.pp.highly_variable_genes()`) only cell-type specific genes (Pangalo [3] ubiquitousness index ≤ 0.1) were retained for all further analyses. Resulting dataset sizes after preprocessing are reported in Table A.1. The filtering steps are stricter than for standard analyses in order to ensure only genes with sufficient evidence for expression are included. For GRN inference, the resulting expression values were additionally scaled to zero mean and unit variance.

The analysis setting for scRNA-seq data mirrored the experiments described for bulk data in the main manuscript. The preprocessed data matrix was used as input to GRNBoost2 for GRN inference. To asses approximation performance, edge-wise empirical P-values were computed using the DIANE-like approach, which was treated as ground truth. The accuracy of SignifiKANTE's approximate P-values across varying numbers of target clusters $\ell \in \{1, \ldots, 10, 20, \ldots, 100\}$ was subsequently evaluated relative to these reference values. Additionally, the number of permutations k for approximate empirical P-value computation was varied, with $k \in \{1000, 3000, 5000, 7000, 10000\}$.

Table A.1. Sizes of scRNA-seq datasets after preprocessing.

Tissue	# Cells	# Genes
Dendritic cells	405	1176
Natural killer cells	613	1176
T cells (CD8+)	347	1176

B Supplementary figures and tables

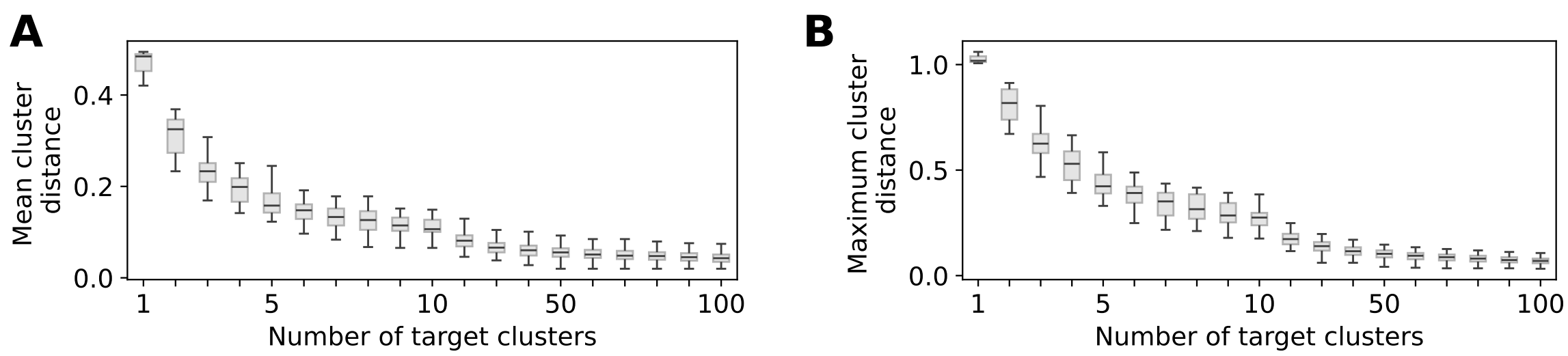

Fig. B.1. Distributions of mean and maximum pairwise intra-cluster 1-Wasserstein distances resulting from 1-Wasserstein distance-based hierarchical clustering of gene expression profiles for three scRNA-seq datasets.

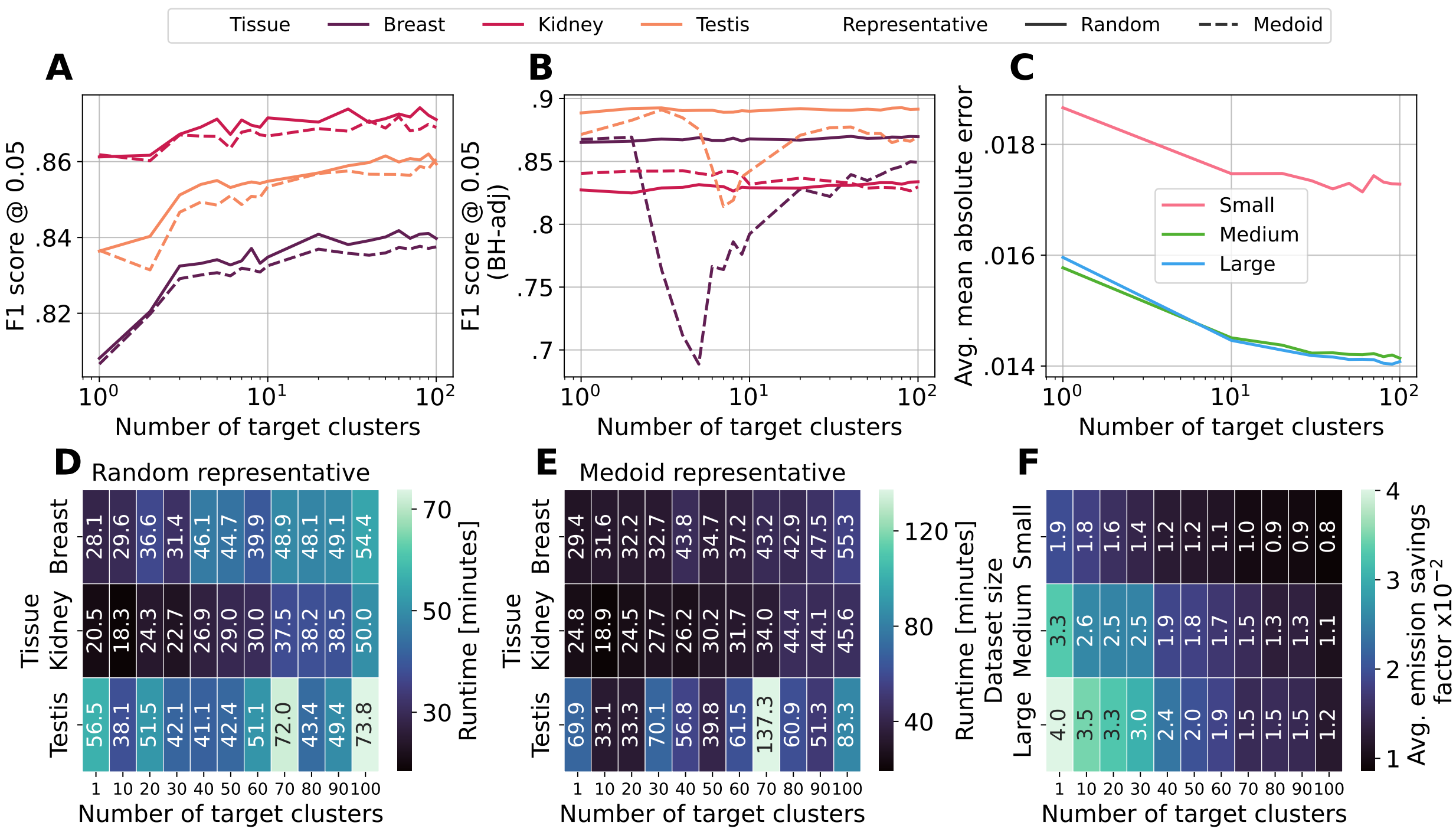

Fig. B.2. Additional results for GTEx data for SignifiKANTE with GRNBoost2 [7] as core GRN inference method. **(A, B, D, E)** Effect of using cluster medoids or randomly sampled cluster genes as cluster representatives. **(C)** Mean absolute errors of SignifiKANTE's approximate P-values against ground truth P-values, averaged over all tissues within the tissue bins "small", "medium", and "large". **(F)** Saved carbon emission in comparison to P-value computation with DIANE-like permutation approach.

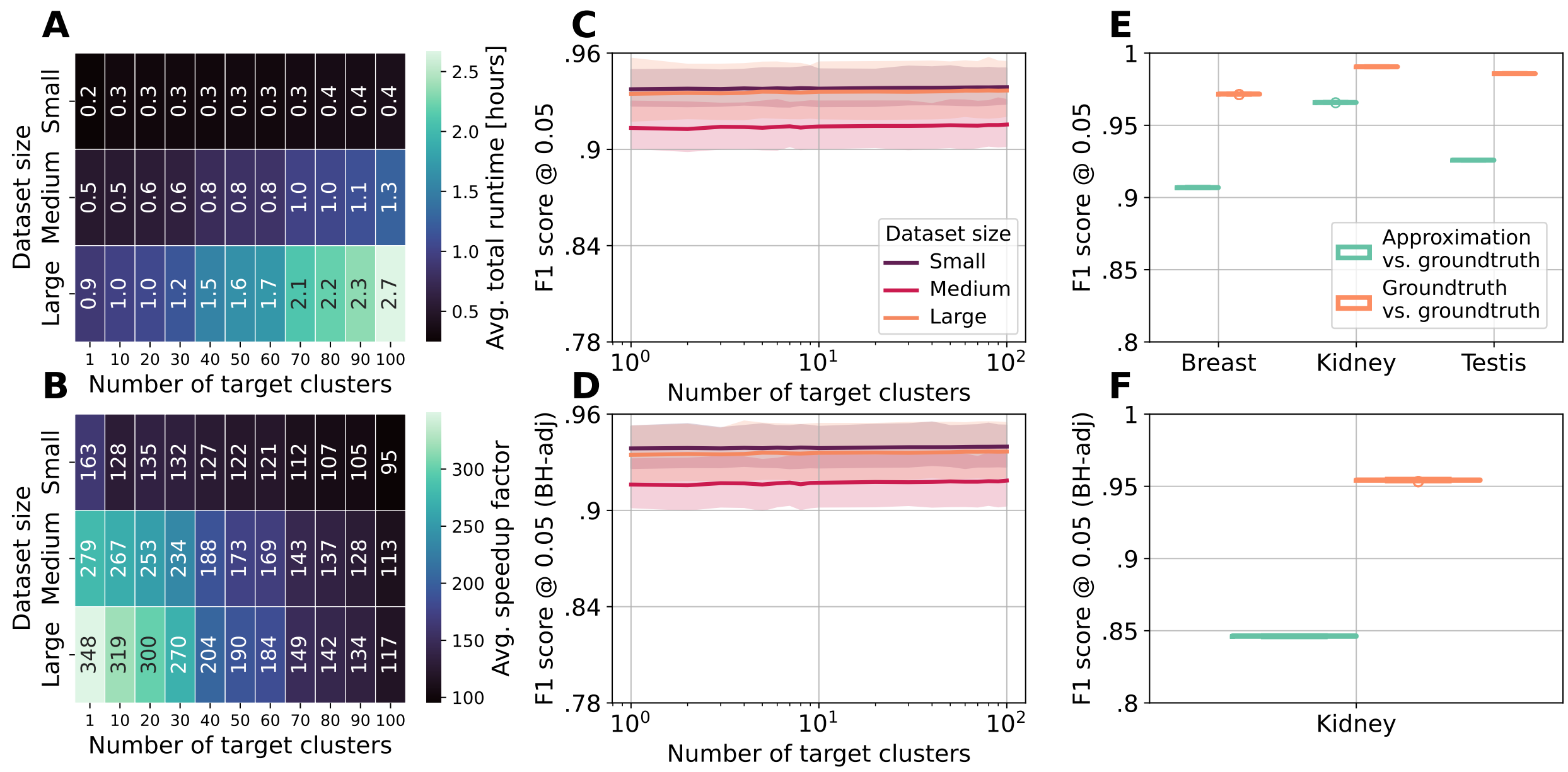

Fig. B.3. Comparison of SignifiKANTE with GReNaDIne's [8] LASSO regression as core GRN inference method to exact empirical P-value computation for GRNs as implemented in DIANE, with $k = 1000$ permutations for both the exact and the approximate empirical P-values. **(A)** Mean SignifiKANTE runtimes across small ($n < 500$), medium ($500 \leq n \leq 1000$), and large ($n > 1000$) GTEx datasets for varying ℓ. **(B)** Corresponding mean speed-up factors in comparison to exact P-value computation. **(C, D)** F1 scores for varying ℓ obtained via comparison of edges in GRNs with significant exact and approximate P-values at 0.05 significance level, both without (C) and with (D) BH correction. In (D), we have excluded tissues for which no edges remained significant in the groundtruth GRN. **(E, F)** F1 scores measuring approximation quality of SignifiKANTE's P-values against ten groundtruth P-values and of ten groundtruth P-value runs against themselves for significance level 0.05, both without (E) and with (F) BH correction. In (F), the breast and testis tissues are excluded, as no edges remained significant after BH correction in the corresponding groundtruth GRNs, likely because the uncorrected P-values' resolution of 1/1001 was too coarse for BH correction. Overall, the results for LASSO backend are very similar to the results for the GRNBoost2 backend shown in Fig. 2 of the main article. The only real difference is that the approximation quality of SignifiKANTE's P-values is no longer optimal up to variation in the computation of the exact permutation-based P-values and the inherent stochasticity of the GRN inference method, as the "groundtruth vs. groundtruth" F1 scores are larger for the LASSO backend than for the GRNBoost2 backend (E, F). A likely reason for this is that GReNaDIne's LASSO regression model is more deterministic than GRNBoost2's boosted tree model.

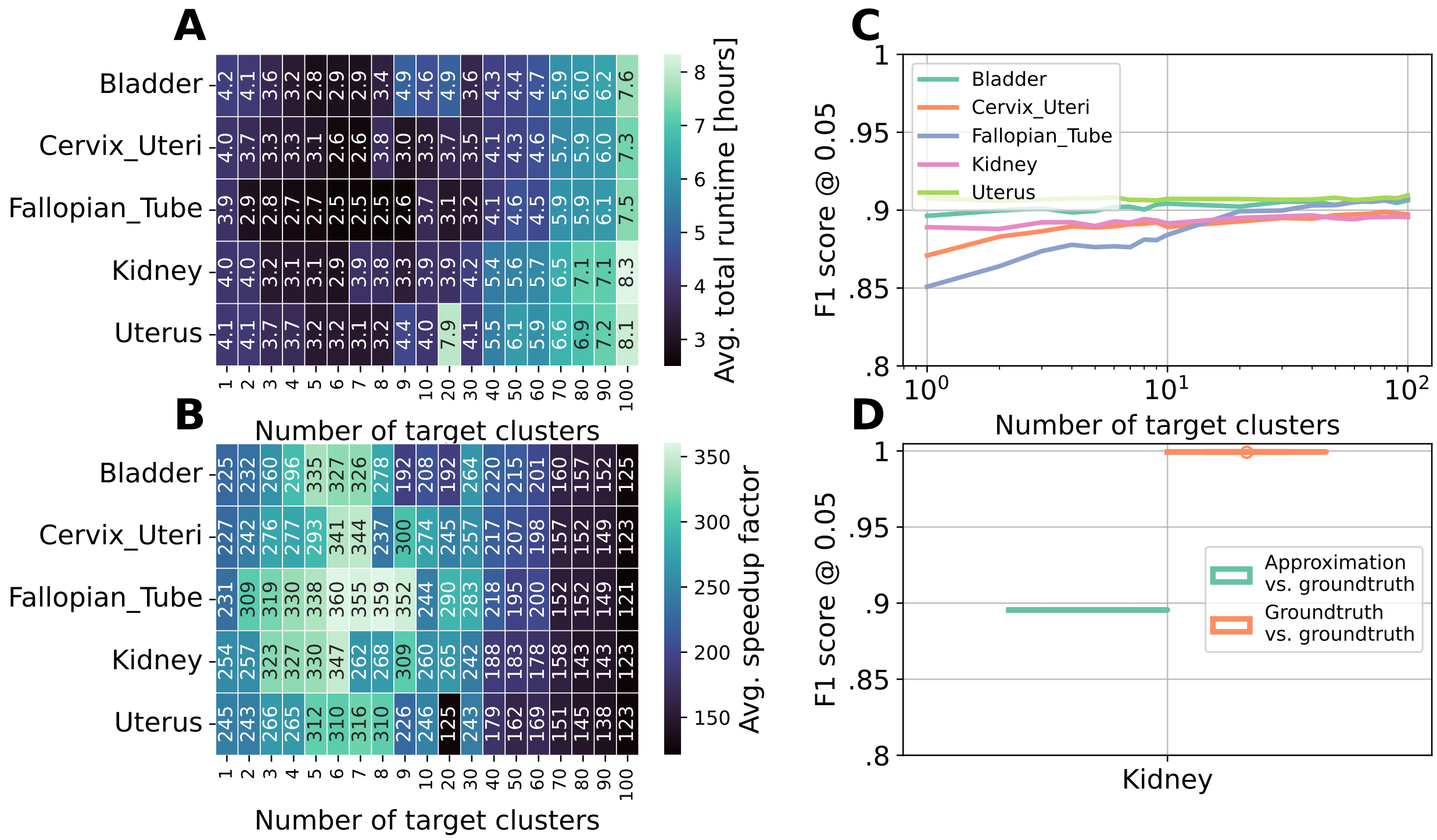

Fig. B.4. Comparison of SignifiKANTE with GReNaDIne's [8] ridge regression as core GRN inference method to exact empirical P-value computation for GRNs as implemented in DIANE, with $k = 1000$ permutations for both the exact and the approximate empirical P-values. Due to substantial runtime requirements of the ridge regression model, tests were run only for five small GTEx tissue datasets. Under BH correction none of the ground truth edges remained significant, which is why we only show results without P-value correction in this plot. **(A)** Mean SignifiKANTE runtimes across five GTEx datasets for varying ℓ. **(B)** Corresponding mean speed-up factors in comparison to exact P-value computation. **(C)** F1 scores for varying ℓ obtained via comparison of edges in GRNs with significant exact and approximate P-values at 0.05 significance level. **(E)** F1 scores measuring approximation quality of SignifiKANTE's P-values against ten groundtruth P-values and of ten groundtruth P-value runs against themselves for significance level 0.05. Results are shown for only one tissue do to the immense compute load of inferring ten groundtruths. Results for BH-corrected P-values are not shown because all groundtruth P-values were non-significant at significance level 0.05 after BH correction, likely because the uncorrected P-values' resolution of 1/1001 was too coarse for BH correction. Consistent with the results for the LASSO regression backend (Figure B.3) and in contrast to the results for the GRNBoost2 backend, the approximation quality of SignifiKANTE's P-values is no longer optimal up to variation in the computation of the exact permutation-based P-values and the inherent stochasticity of the GRN inference method, as the "groundtruth vs. groundtruth" F1 scores for the ridge regression backend are near-perfect (D). A likely reason for this is that GReNaDIne's ridge regression model is more deterministic than GRNBoost2's boosted tree model.

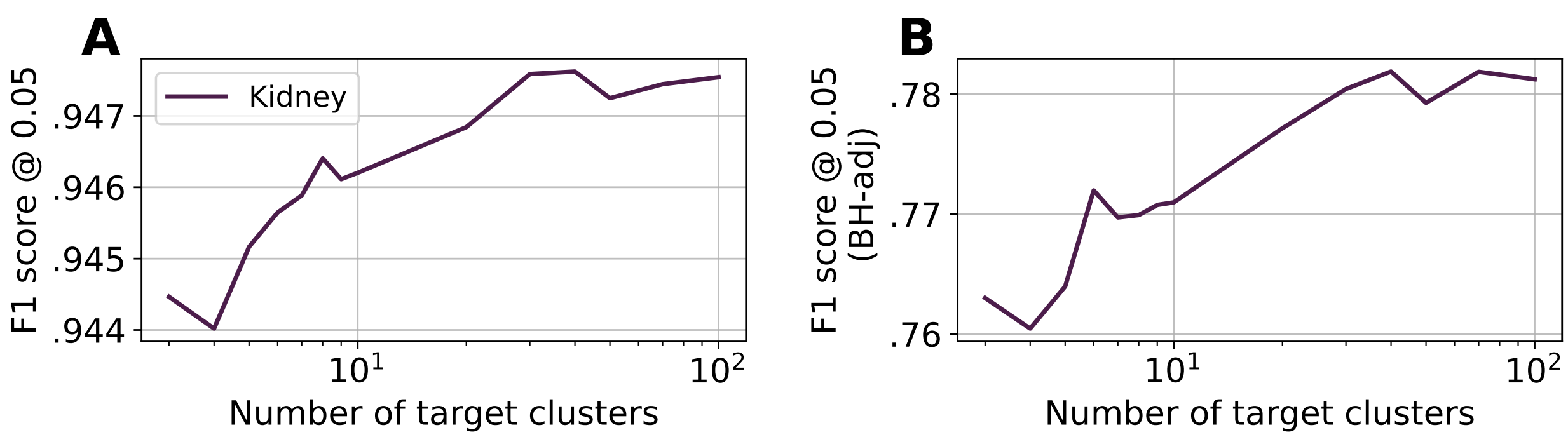

Fig. B.5. Comparison of SignifiKANTE with GENIE3 [6] as core GRN inference method to exact empirical P-value computation for GRNs as implemented in DIANE. Due to substantial runtime requirements of GENIE3, tests were run only for the kidney tissue dataset. **(A, B)** F1 scores for varying ℓ obtained via comparison of edges in GRNs with significant exact and approximate P-values at 0.05 significance level, both without (A) and with (B) BH correction.

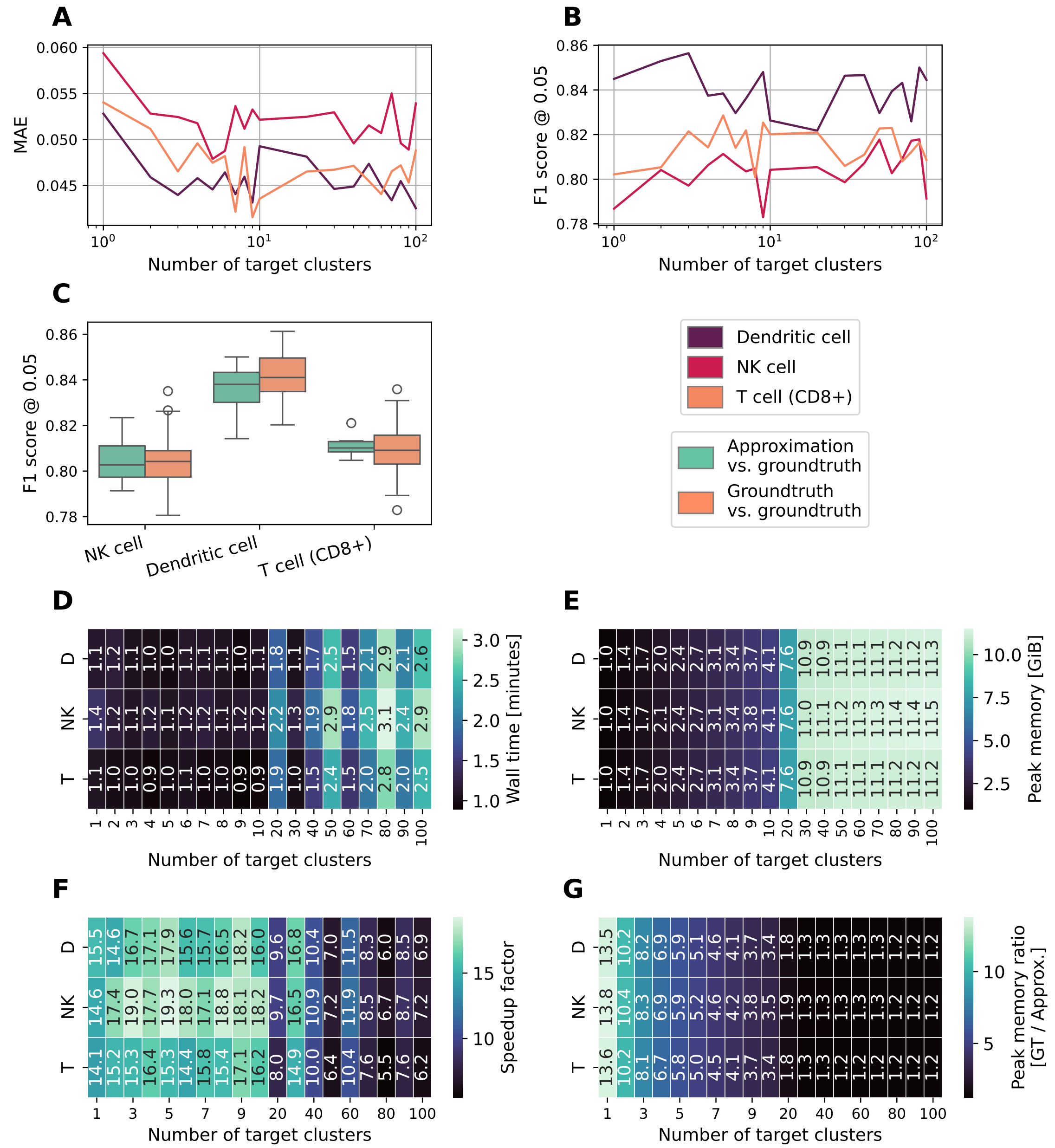

Fig. B.6. Results obtained with SignifiKANTE with (GRNBoost2 as core GRN inference method, $k = 1000$ permutations) for three scRNA-seq datasets of dendritic cells, natural killer cells, and CD8-positive T cells, respectively. **(A, B)** Comparison of uncorrected approximate P-values to DIANE-like exact empirical P-values as a function of the number of target clusters used for the approximation, quantified by mean absolute error (A) and F1 score at significance level 0.05 (B). Both metrics converge and remain stable for more than 10 target clusters. **(C)** F1 scores at significance threshold 0.05 generated by pairwise comparison of 10 ground truth runs and comparison between a single approximation run ($\ell = 100$ target clusters) and each ground truth run. The similarity of F1 scores across both comparison settings suggests that, also for scRNA-seq data, SignifiKANTE's approximations is optimal up to variation in the computation of the exact permutation-based P-values. **(D, E)** Absolute values of wall-clock time (D) and peak memory (E) for the approximate computation across different numbers of target clusters. Peak memory does not increase beyond the point where the number of target clusters is equal to or larger than the number of CPUs allocated for the computation (here: 30 CPUs), showing that the parallelization with Dask induces a memory overhead. **(D, E)** Corresponding speed-up fractions (F) and peak memory ratios (G). Results for BH-corrected P-values are not shown because all groundtruth P-values were non-significant at significance level 0.05 after BH correction, likely because the uncorrected P-values' resolution of 1/1001 was too coarse for BH correction.

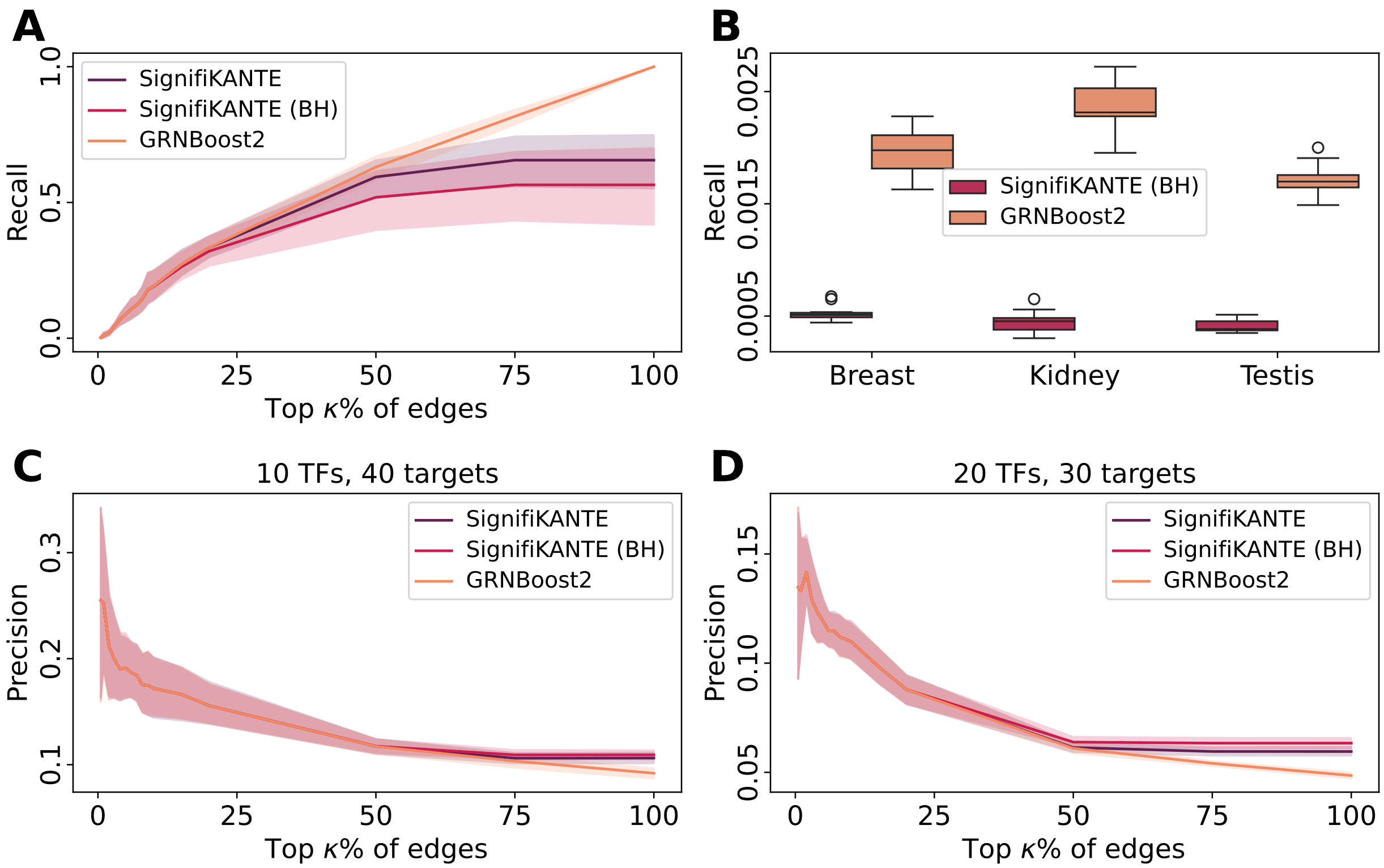

Fig. B.7. Additional results for simulated and real-world data. **(A)** Recall of top $\kappa\%$ edges (sorted by weights) inferred from simulated expression data against groundtruth GRN used for simulation, using all GRNBoost2 edges, all significant edges of SignifiKANTE (significance cutoff at $\alpha = 0.05$, $\ell = 10$ target gene clusters), and all significant edges of SignifiKANTE with BH correction (FDR control at $q = 0.05$, $\ell = 10$). **(B)** Distributions of recall values between all GRNBoost2 edges inferred from breast, kidney, and testis GTEx data and only significant edges computed by SignifiKANTE (BH correction, and FDR control at $q = 0.05$, $\ell = 100$). Known TF-target interactions from the CollecTRI database [19] were treated as groundtruth. Using different random seeds, we ran GRNBoost2 followed by SiginiKANTE ten times on the same data. **(C, D)** Additional precision values on simulated expression data, for the setting of 10 TFs with 40 targets each (C), and the setting of 20 TFs with 30 targets each (D).

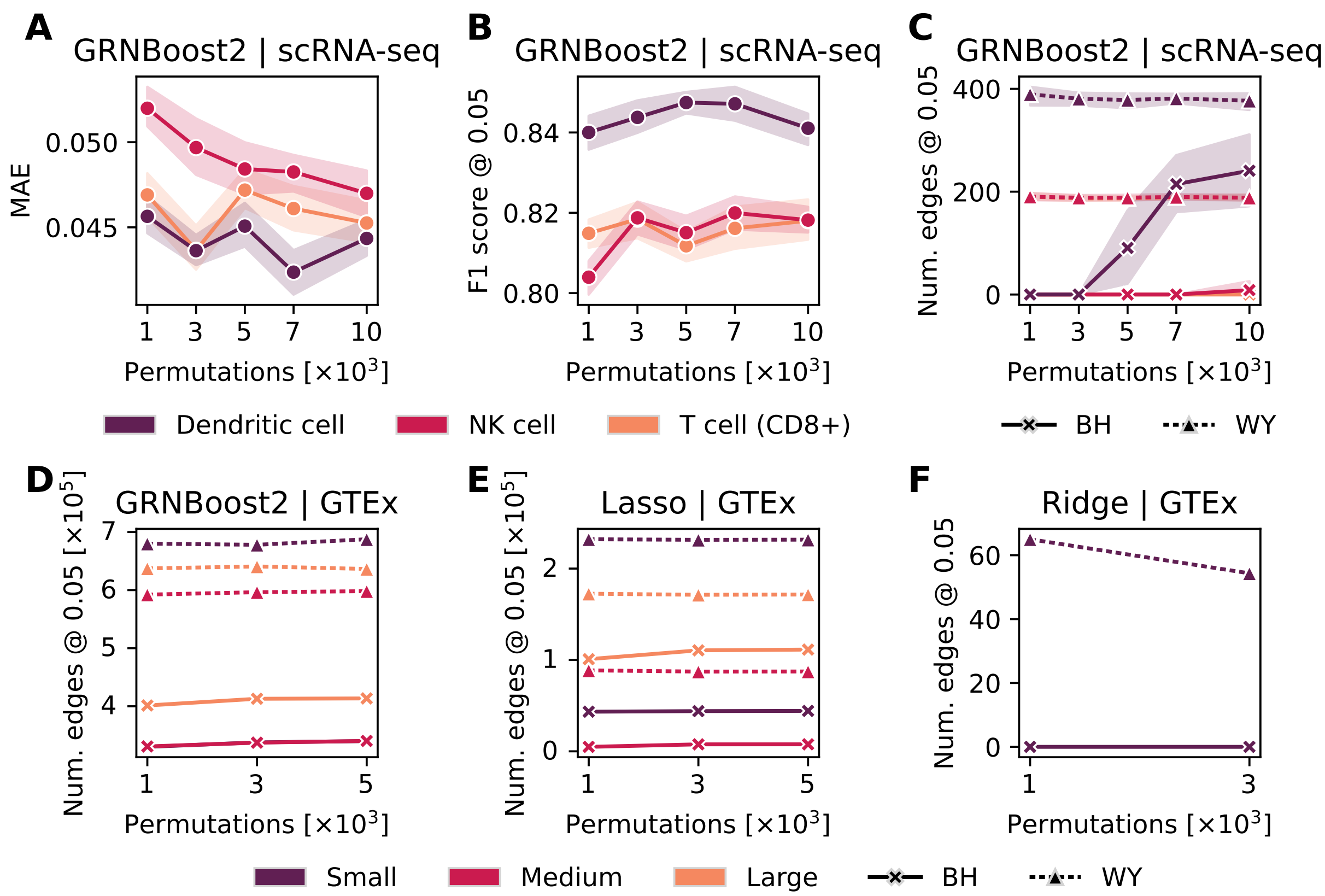

Fig. B.8. Effect of the number of permutations k and the multiple testing correction method on SignifiKANTE, evaluated on the scRNA-seq datasets and bulk RNA-seq datasets from GTEx. **(A, B)** Mean absolute errors and F1 scores at significance level 0.05 for uncorrected approximate P-values and varying k, using uncorrected DIANE-like P-values computed with the same k as reference. Due to runtime constraints, we carried out this ablation study only for the scRNA-seq datasets, as running the exact DIANE-like approach with $k > 1000$ permutations for several k on the GTEx datasets was computationally infeasible. **(C–F)** Number of significant edges after multiple testing correction using Benjamini-Hochberg correction (BH, FDR control at $q = 0.05$) and Westfall-Young correction (WY, target gene cluster-level FWER control at $q = 0.05$). For (A–C), reported values are averaged over varying numbers of target clusters $\ell \in \{1, \ldots, 10, 20, \ldots, 100\}$. For (D, E), values are averaged over the 30 GTEx tissues, categorized into small, medium, and large by their number of samples. For (F), values are averaged over five GTEx tissues with small sample sizes due to runtime constraints. Shaded regions indicating empirical 95% confidence intervals. Note that in (C), the values for NK cells (red) closely match those for CD8+ T cells (orange), such that the CD8+ T cell curve is nearly completely masked by the NK cell curve. Similarly, in (D), the curve showing the number of significant edges after BH correction for small tissues (purple) is almost completely covered by the corresponding curve for medium-sized tissues (red).

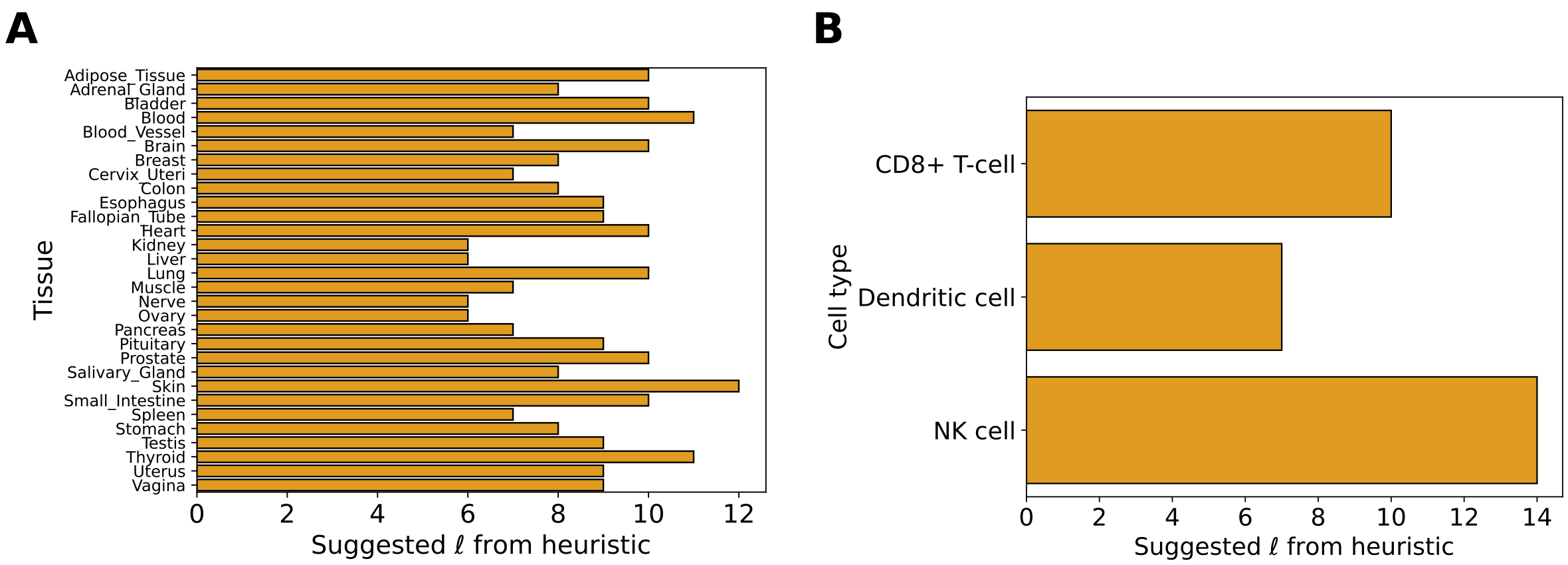

Fig. B.9. Suggested number of clusters ℓ from the heuristic `find_optimal_number_of_clusters` implemented in SignifiKANTE on all thirty GTEx tissue datasets (**A**) and three single-cell datasets (**B**).

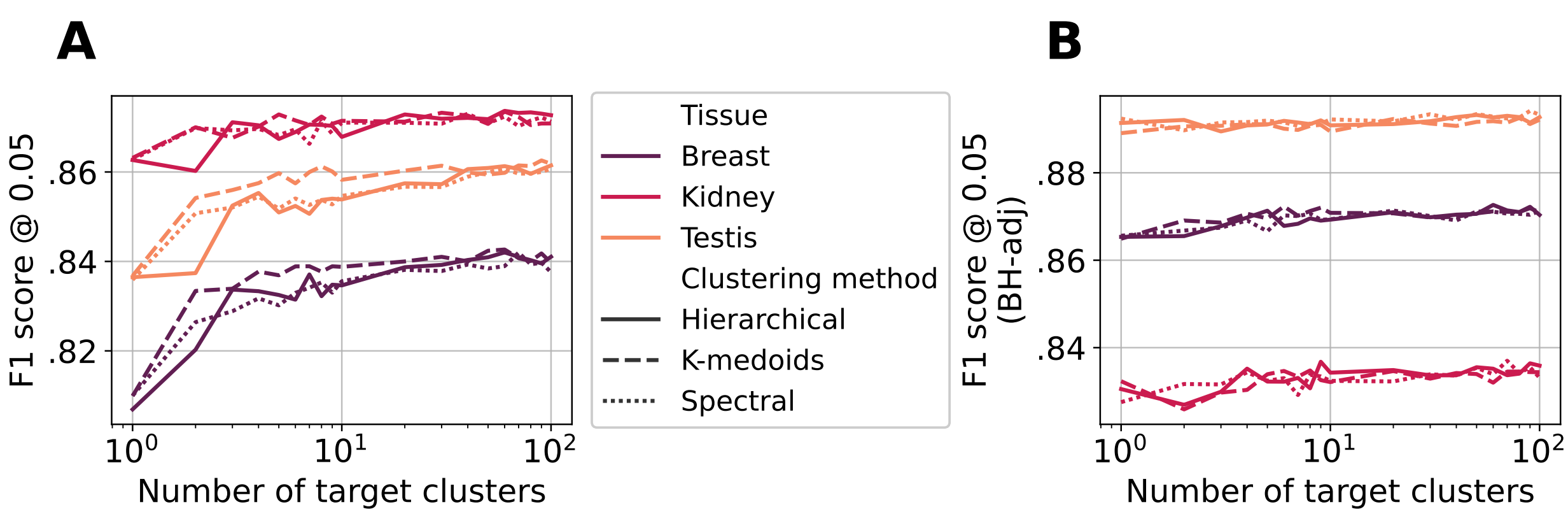

Fig. B.10. Comparison of different clustering algorithms used within SignifiKANTE on three GTEx tissue datasets. As default, SignifiKANTE employs hierarchical clustering on the Wasserstein distance matrix ("Hierarchical"). Additionally, we compared this to using K-medoids clustering ("K-medoids") and spectral clustering ("spectral") on the Wasserstein distance matrix. F1 scores are shown with (**A**) and without (**B**) BH correction on P-values.

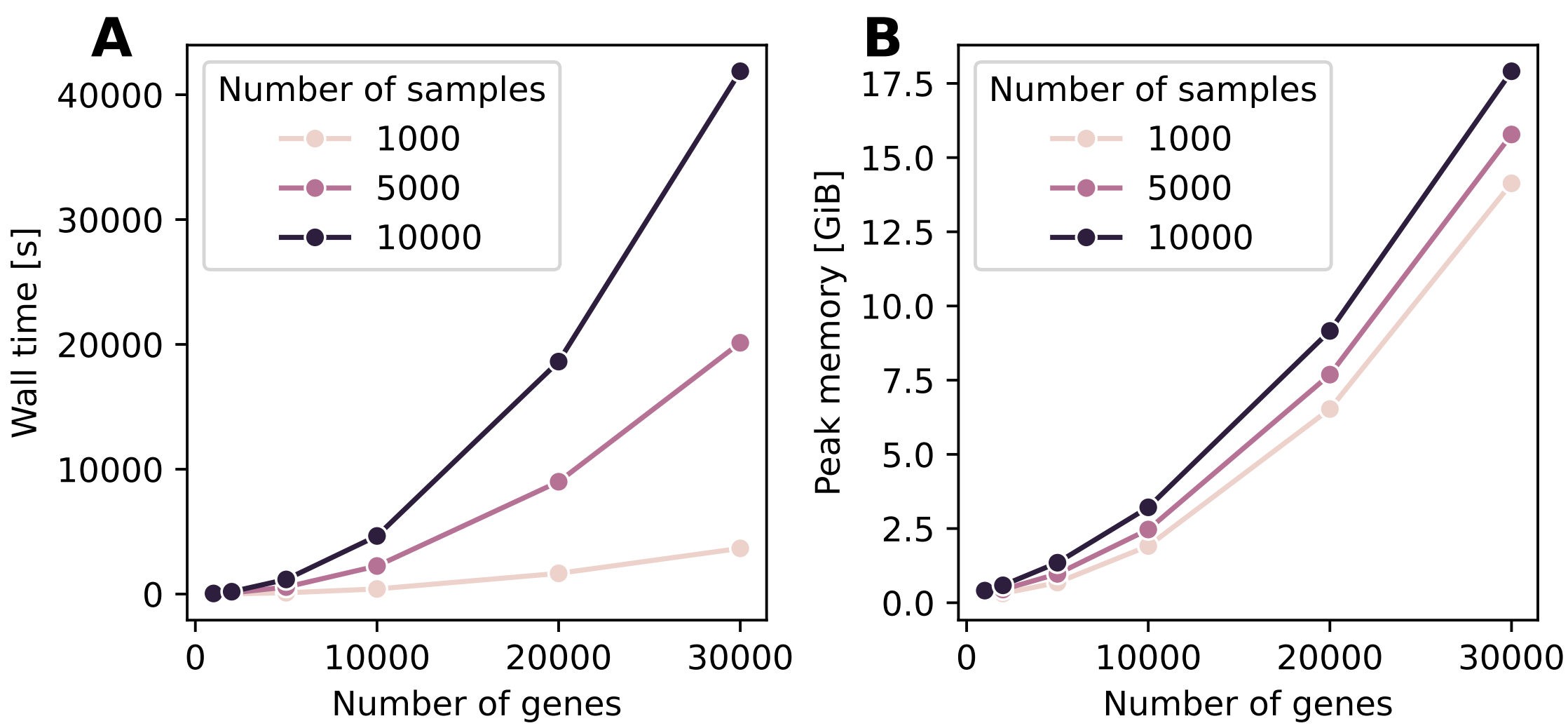

Fig. B.11. Benchmark of Wasserstein distance matrix computation showing wall-clock time in seconds **(A)** and peak memory usage in gibibyte **(B)** as a function of the number of genes for different numbers of samples ($n \in \{1000, 5000, 10000\}$). Benchmarks were performed on dense, non-sparse data matrices with entries sampled uniformly at random. Shown values are averages across 10 trials.

Table B.1. Overview of GTEx data, including the number of samples and genes after preprocessing per tissue, and the number of edges and significant edges in the reference GRN computed by GRNBoost2. Significant edges for each tissue are computed using SignifiKANTE's P-values with $\ell = 100$, BH correction, and a significance cutoff of 0.05. The significance ratio is computed as the ratio of significant edges and the number of total edges.

Tissue	# Samples	# Genes	# Edges	# Significant edges	Significance ratio
Adipose_Tissue	1204	15620	1540796	340828	0.2212
Adrenal_Gland	258	15630	1891813	307457	0.1625
Bladder	21	15864	2304629	246183	0.1068
Blood	929	13896	1724486	322745	0.1872
Blood_Vessel	1335	15313	1501239	307109	0.2046
Brain	2642	15884	2022744	608754	0.3010
Breast	459	15755	1746068	285476	0.1635
Cervix_Uteri	19	15672	2236599	190448	0.0851
Colon	779	15888	1769981	354494	0.2003
Esophagus	1445	15538	1765118	388435	0.2201
Fallopian_Tube	9	15738	1947223	1063782	0.5463
Heart	861	15216	1549753	446156	0.2879
Kidney	89	16030	2079742	335425	0.1613
Liver	226	15282	1818300	271637	0.1494
Lung	578	16091	1751109	282877	0.1615
Muscle	803	14852	1551872	282176	0.1818
Nerve	619	15975	1694849	305257	0.1801
Ovary	180	15599	2006770	205126	0.1022
Pancreas	328	15606	1900876	420292	0.2211
Pituitary	283	16448	1983108	287424	0.1449
Prostate	245	16210	1977624	285565	0.1444
Salivary_Gland	162	16123	2019478	287912	0.1426
Skin	1809	15120	1994850	399343	0.2002
Small_Intestine	187	16246	2067232	298896	0.1446
Spleen	241	15945	1913360	315616	0.1649
Stomach	359	15769	1902568	285085	0.1498
Testis	361	18124	2049277	424118	0.2069
Thyroid	653	15993	1711783	320693	0.1873
Uterus	142	15681	2084537	179294	0.0860
Vagina	156	15878	2135811	265521	0.1243

Table B.2. Mapping of GTEx [5] tissue names to terms in the BRENDA tissue ontology (BTO) [4]. The mapping was manually generated using semantic similarity between the terms.

GTEx tissue name	BTO term	GTEx tissue name	BTO term
Adipose_Tissue	BTO:0001487	Adrenal_Gland	BTO:0000047
Bladder	BTO:0001418	Blood	BTO:0000089
Blood_Vessel	BTO:0001102	Brain	BTO:0000142
Breast	BTO:0000149	Cervix_Uteri	BTO:0002249
Colon	BTO:0000269	Esophagus	BTO:0000959
Fallopian_Tube	BTO:0000980	Heart	BTO:0000562
Kidney	BTO:0000671	Liver	BTO:0000759
Lung	BTO:0000763	Muscle	BTO:0000887
Nerve	BTO:0000925	Ovary	BTO:0000975
Pancreas	BTO:0000988	Pituitary	BTO:0001073
Prostate	BTO:0001129	Salivary_Gland	BTO:0001203
Skin	BTO:0001253	Small_Intestine	BTO:0000651
Spleen	BTO:0001281	Stomach	BTO:0001307
Testis	BTO:0001363	Thyroid	BTO:0001379
Uterus	BTO:0001424	Vagina	BTO:0000243

References

1. Cassan, O., Lèbre, S., Martin, A.: Inferring and analyzing gene regulatory networks from multi-factorial expression data: a complete and interactive suite. BMC Genomics **22**(1), 387 (2021). https://doi.org/10.1186/s12864-021-07659-2
2. De Simone, M., Hoover, J., Lau, J., Bennett, H., Wu, B., Chen, C., Menon, H., Au-Yeung, A., Lear, S., Vaidya, S., Shi, M., Lund, J., Xavier-Magalhães, A., Liang, Y., Kurdoglu, A., O'Gorman, W., Modrusan, Z., Le, D., Darmanis, S.: A comprehensive analysis framework for evaluating commercial single-cell rna sequencing technologies. Nucleic Acids Res. **53**(2), gkae1186 (2025). https://doi.org/10.1093/nar/gkae1186
3. Franzén, O., Gan, L.M., Björkegren, J.L.M.: Panglaodb: a web server for exploration of mouse and human single-cell rna sequencing data. Database **2019**, baz046 (2019). https://doi.org/10.1093/database/baz046
4. Gremse, M., Chang, A., Schomburg, I., Grote, A., Scheer, M., Ebeling, C., Schomburg, D.: The BRENDA tissue ontology (BTO): the first all-integrating ontology of all organisms for enzyme sources. Nucleic Acids Res. **39**(Database issue), D507–13 (2011). https://doi.org/10.1093/nar/gkq968
5. GTEx Consortium: The genotype-tissue expression (GTEx) project. Nat. Genet. **45**(6), 580–585 (2013). https://doi.org/10.1038/ng.2653
6. Huynh-Thu, V.A., Irrthum, A., Wehenkel, L., Geurts, P.: Inferring regulatory networks from expression data using tree-based methods. PLoS One **5**(9), e12776 (2010). https://doi.org/10.1371/journal.pone.0012776
7. Moerman, T., Aibar Santos, S., Bravo González-Blas, C., Simm, J., Moreau, Y., Aerts, J., Aerts, S.: GRNBoost2 and Arboreto: efficient and scalable inference of gene regulatory networks. Bioinformatics **35**(12), 2159–2161 (2019). https://doi.org/10.1093/bioinformatics/bty916
8. Schmitt, P., Sorin, B., Frouté, T., Parisot, N., Calevro, F., Peignier, S.: GReNaDIne: A data-driven python library to infer gene regulatory networks from gene expression data. Genes (Basel) **14**(2), 269 (2023). https://doi.org/10.3390/genes14020269
